# Unraveling G‐Quadruplex and i‐Motif Coexistence Within a Double‐Stranded DNA

**DOI:** 10.1002/anie.3958607

**Published:** 2026-06-01

**Authors:** Davide Auricchio, Michele Ghezzo, Uroš Zavrtanik, Luca Bertini, Valeria Libera, Riccardo Rigo, Jurij Lah, Claudia Sissi

**Affiliations:** ^1^ Department of Pharmaceutical and Pharmacological Sciences University of Padova Padova Italy; ^2^ Department of Physical Chemistry Faculty of Chemistry and Chemical Technology University of Ljubljana Ljubljana Slovenia; ^3^ Department of Physics and Geology University of Perugia Perugia Italy

**Keywords:** circular dichroism, DSC, G‐quadruplex, i‐Motif, SAXS

## Abstract

DNA can transiently fold into variable arrangements, which are expected to exploit regulatory functions. Guanine‐rich sequences can fold into G‐quadruplexes (G4s), while the complementary strand adopts potentially i‐Motif (iM) arrangements. Their concomitant formation at the same genomic site is still under debate. However, recently, single‐molecule analyses have shown the simultaneous G4 and iM presence within a double‐stranded (ds) DNA context, addressing them as synergic blockers of replication fork progression. While these findings point to a functional interplay between G4 and iM, a deeper understanding of the factors enabling their coexistence remains unclear. In this work, we unravel the equilibria governing G4‐ and iM‐folding within dsDNA, adopting an extensive biophysical approach allowing analysis of an optimized modular system, scalable across constructs of increasing molecular complexity. Our findings corroborate the simultaneous formation model and further clarify the thermodynamic determinants driving duplex denaturation and the favorable folding of stable G4 and iM structures.

## Graphical abstract

The relative thermodynamic profiles of G‐quadruplex (G4), i‐Motif (iM), and the duplex counterpart tune the simultaneous G4 and iM formation within a double‐stranded (ds) DNA.

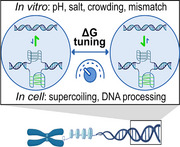

## 1 Introduction

Within the cell, DNA folding continuously rearranges from the prevalent *B*‐helix toward unwound helices, single‐stranded, up to various noncanonical structures [1, 2, 3]. Some of them cluster at genomic sites that share unique base composition. Among them, guanine‐rich regions are those more extensively studied due to their potential folding into G‐quadruplex structures (G4), tetra‐helical arrangements driven by the pairing of four guanines through Hoogsteen hydrogen bonds [4]. The stacking of these planar G‐quartets and their coordination of monovalent cations (K^+^ or Na^+^) further contribute to the G4 stability and, along with the length and base composition of the connecting loops, to define the preferred topology [4, 5].

At most G4‐forming sites, the complementary C‐rich strand can potentially fold into i‐Motif (iM), antiparallel tetra‐helical structures held together by intercalated hemi‐protonated C:C^+^ base pairs [6, 7]. Since cytosine *pK*_a_ is ≈4.6, originally iMs were not considered as physiologically relevant. However, in vitro, by increasing the number of C:C^+^ base pairs and/or by applying molecular crowding conditions to simulate the nuclear environment, it was possible to observe iM formation by C‐rich genomic sequences also at pH close to the physiological one [8, 9, 10, 11]. Recently, different genome‐wide experimental approaches succeeded in mapping iM *in cell* [12, 13]. As well, the genomic distribution of functional G4s has been extensively explored in various cellular models [14, 15, 16]. From these studies, the occurrence of iM and G4 at overlapping sites emerged, thus suggesting that both these structural modules can work as regulatory elements of related biological processes, either individually or through fine‐tuning of protein recruitment [13, 17, 18].

Although according to the sequence requirements, G4s and iMs can form at complementary strands, their concomitant formation when these sites are embedded within a double‐stranded (ds) DNA environment is still under investigation. Indeed, unwinding appeared to be required to reduce the high stability of the competing B‐helix at GC‐rich sites [19, 20, 21, 22, 23, 24]. In addition, early biochemical and single‐molecule studies reported simultaneous G4‐ and iM‐folding only when the two tetra‐helices were located out‐of‐phase at a proper distance along the complementary strands, pointing to their mutual exclusion due to steric hindrance [25, 26, 27]. Consistently, immunostaining data suggested their interdependent formation at distinct cell cycle phases and related G4s and iMs to gene expression activation and repression, respectively [28].

Conversely, iM mapped in proximity to G4‐forming regions at genes with high transcription rates, and at those expressed in G0/G1 phase, in line with functional studies that supported similar regulatory functions for both these secondary structures [29, 30, 31, 32, 33, 34]. In addition, although *in cell* NMR data showed iM‐folding at a restricted fraction of the predicted sites, they also validated the positive G4‐iM physiological interplay resulting from their simultaneous formation at the telomeric level [35, 36, 37, 38]. Very recently, Sun and coworkers, by means of single‐molecule techniques, provided solid evidence about the coexistence of G4 and iM within a promoter ds DNA domain at neutral pH, and, from the analyses of the mechanical stability of the tested construct, they confirmed a mutual influence of the two noncanonical structures [39]. This model was used to monitor the progression of the replication fork and confirmed a coordinate interplay between the two structures. However, in contrast to most physiological processes, the G4‐/iM‐unfolding of this model was nearly irreversible. This also prevented a comprehensive thermodynamic characterization of the different species occurring along the folding/unfolding steps, which is required to rationalize the driving forces that regulate such an interplay at different sites and within different genomic environments (i.e., single strand (ss), ds, and unwound DNA).

**FIGURE 1:**
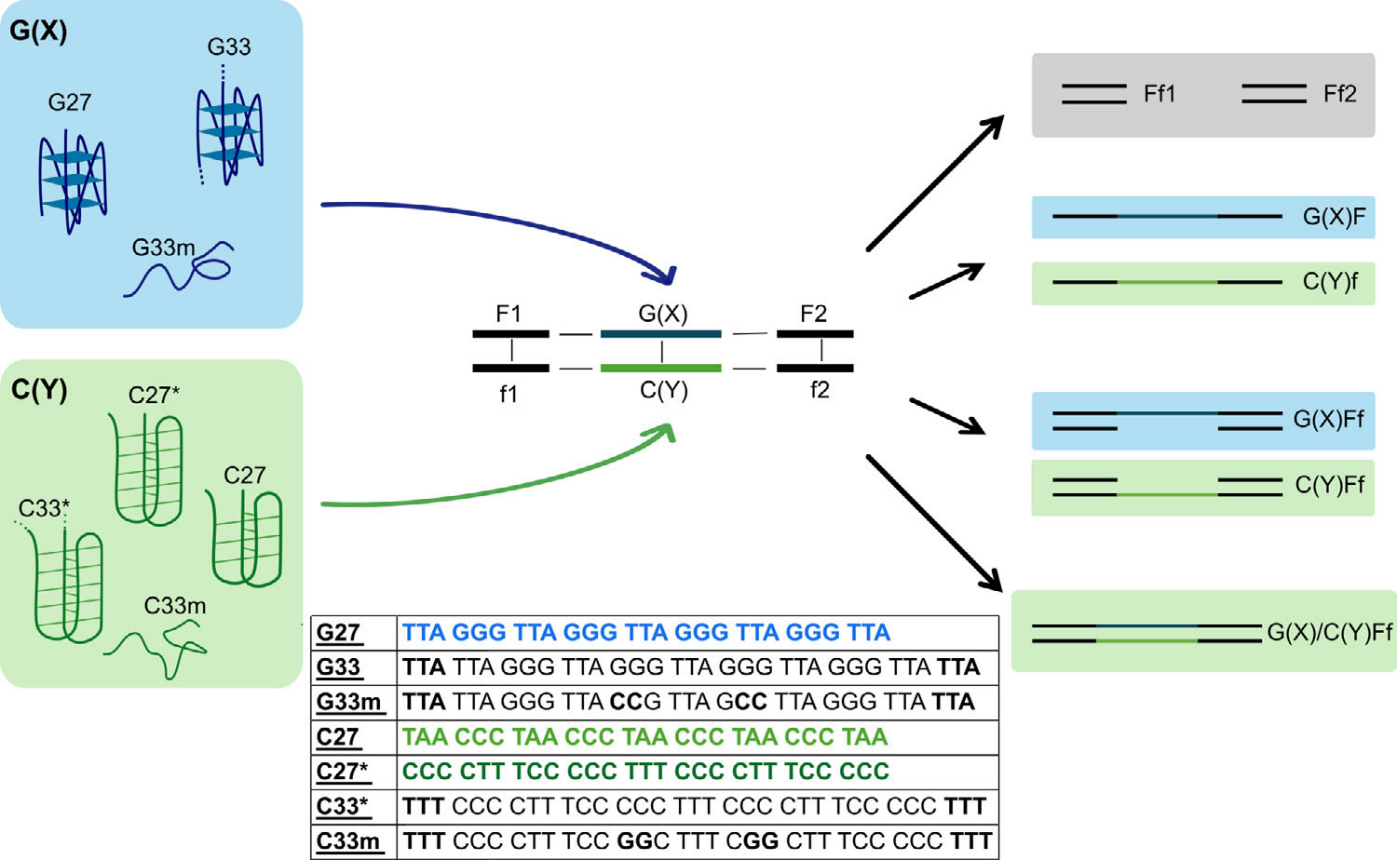
Schematic representation of the modular structural construct used in this study. The sequences of the selected G‐ and C‐rich domains that differ in length (27 or 33 nts) and composition (27*, 33*, and 33 m)—overall summarized as G(X) and C(Y)—are reported in the central panel. Residues in bold indicate sequence modifications. Ff1 and Ff2 are pairs of fully complementary sequences added at the terminals of the G(X) and C(Y) strands. The complete list of all tested sequences is reported in Table S1.

To fill this gap, we developed a modular system that was optimized in a stepwise manner, using an integrated suite of spectroscopic, electrophoretic, calorimetric, and scattering techniques, including UV absorption spectroscopy (UV), circular dichroism (CD), polyacrylamide gel electrophoresis (PAGE), differential scanning calorimetry (DSC), and small‐angle x‐ray scattering (SAXS). This design was inspired by our previous work in which single‐molecule FRET was used to elucidate the influence of ds flanking domains on the G4‐folding pathway of two ss G‐rich sequences from the cKIT promoter (c‐kit2 and c‐kit*) [40]. Here, to unravel the structural equilibria among G4, iM, and ds within a genomic‐like context, we embedded the telomeric G‐rich sequence (G(X)) paired to a C‐rich one (C(Y)), within the previously used ds flanking domains (Ff1 and Ff2) that preserved their canonical pairing regardless of the central domain composition (Figure 1). Subsequently, we modified the central segment to meet specific requirements that emerged during the project development. They involved both nucleotide length (27 or 33 nt) and sequence composition (27, 27*, 33*, and 33 m), applied to the G‐rich, the C‐rich, or both strands (see Table S1 for the complete list of used sequences). Overall, this allowed us to mimic both a canonical and an unwound B‐DNA, where the reduced helical stability is expected to boost the folding of noncanonical structures. All full‐length constructs and isolated domains have been comprehensively characterized. By merging the acquired results, we addressed at the molecular level the G4/iM co‐localization, providing the thermodynamic parameters that support their simultaneous formation.

## 2 Results and Discussion

### 2.1 From a Fully to a Partially Complementary Construct

To characterize the colocalization of G4/iM within a B‐DNA context, we initially inserted in the central domain of our construct two fully complementary G‐ and C‐rich strands (G27 and C27) for the human telomeric sequence, since they have been thoroughly investigated [41, 42, 43, 44, 45, 46, 47, 48]. Moreover, telomeric G‐rich sequences can fold into different G4 topologies, and we considered it beneficial to avoid structural constraints when the noncanonical structures must fit within the final full‐length construct [49]. Accordingly, we also extended the minimal G4 and iM forming sequences by one telomeric triplet (TTA and AAT at the G‐ and C‐rich strand, respectively) at both terminals. These G27 and C27 sequences were finally flanked by previously used complementary domains (F1 or f2 at 5′ and F2 or f1 at 3′) [40]. Accordingly, we obtained two fully complementary 69 nts‐long oligonucleotides (G27F and C27f) that, once annealed, generated a fully dsDNA (G27/C27Ff, Figure 1).

**FIGURE 2:**
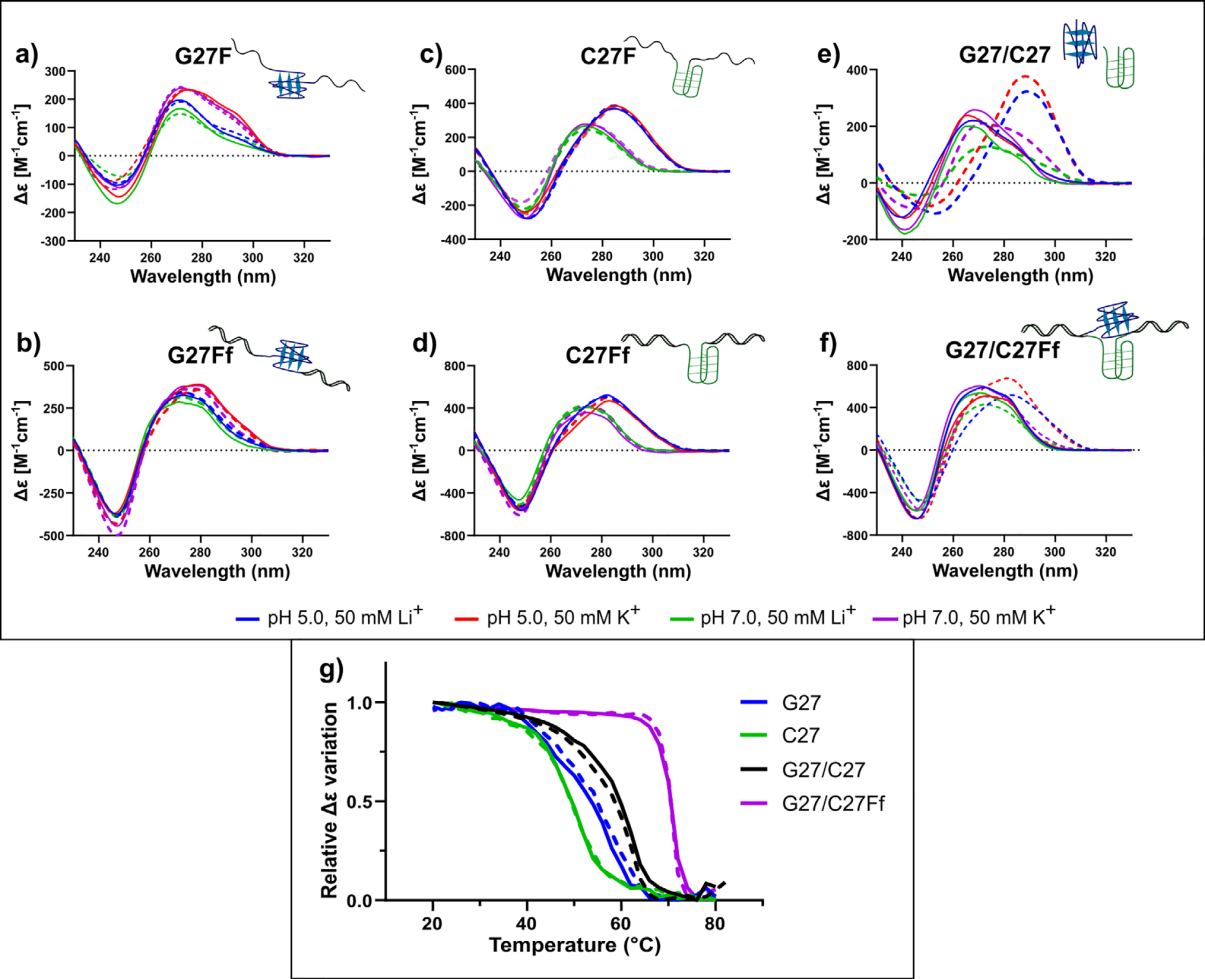
Chiroptical characterization of the fully complementary construct. (a–f) Comparison of the experimentally acquired CD spectra (solid lines) with the linear combination obtained from the reference spectra reported in Figure S1 (dotted lines) for 1 µM G27F (a), G27Ff (b), C27f (c), C27Ff (d), G27/C27 (e) and G27/C27Ff (f) in cacodylate buffer at pH 7.0, with 50 mM KCl (purple lines) or 50 mM LiCl (green lines) and at pH 5.0, with 50 mM KCl (red lines) or 50 mM LiCl (blue lines). (g) Relative variation of the CD signal as a function of the temperature for 3 µM G27 (blue line), 3 µM C27 (green line), 30 µM G27/C27 (black line) and 10 µM G27/C27Ff (violet) recorded at the wavelength of maximal intensity (290, 285, 270 and 248 nm, respectively) in cacodylate buffer, pH 5.0, 50 mM KCl. Solid and dotted lines refer to melting and annealing processes, respectively.

For each segment, both in the ss and ds form, we acquired the CD spectra at pH 7.0 and 5.0 to modulate iM folding and in 50 mM KCl or LiCl to stabilize or destabilize the G4 structure, respectively (Figure S1) [46, 50]. The CD spectra of G27 in 50 mM KCl at pH 7.0 and 5.0 confirmed its folding into a hybrid‐2 G4 with a positive peak at 290 nm and a shoulder at 265 nm, while no G4 signature was detected in 50 mM LiCl (Figure S1) [44, 51]. The iM‐folding of C27 was confirmed by a positive band centered at 285 nm and a negative one at 265 nm, which were lost at neutral pH [52]. Conversely, the CD profiles of the single (F1, F2, f1, and f2) and double (Ff1 and Ff2) stranded flanking domains were conserved across all tested conditions (Figure S1). These data provided us with the reference spectra for every structural domain of our working construct under all tested conditions: whenever the G4, iM, and ds folding were not affected by the nearby co‐presence of multiple structural domains, the CD spectrum of each construct is expected to correspond to the sum of the chiroptical contributions of all the occurring structural components. This allowed us to generate the so‐called predicted chiroptical profiles of each multi‐component construct (G27F, C27f, G27Ff, C27Ff, G27/C27, and G27/C27Ff) (See Method‐Circular Dichroism in Supporting Information) under all selected experimental conditions, and we compared them to those experimentally acquired. The obtained output is summarized in Figure 2. It showed that the addition of ss or ds flanking domains to G27 or C27 did not significantly affect their folding. Conversely, the experimentally acquired CD spectra of the annealed G27/C27 or G27/C27Ff did not match the predicted iM and G4 presence (Figure 2e,f), while they retained the chiroptical signature of a dsDNA of comparable base composition, in line with the reported competition between B‐helix and noncanonical tetra‐helices occurring at fully paired ds sites [20, 23, 39, 53]. To validate this behavior within our system, we acquired the melting profiles of G27 and C27 as ss and ds at pH 5.0, 50 mM KCl, where both iM and G4 can form (Figures 2g and S2). The two ss oligonucleotides showed a single reversible transition with melting temperature (*T*_m_) at 54°C (G27) and 49°C (C27), assigned to the G4‐ and iM‐unfolding, respectively. Also, the fully paired G27/C27 and G27/27CFf showed one thermal transition that, for the longer construct, was shifted to higher temperatures (*T*_m_ 60°C and 69°C, respectively), thereby fully supporting that the central domain was trapped into the canonical B‐helix.

**FIGURE 3:**
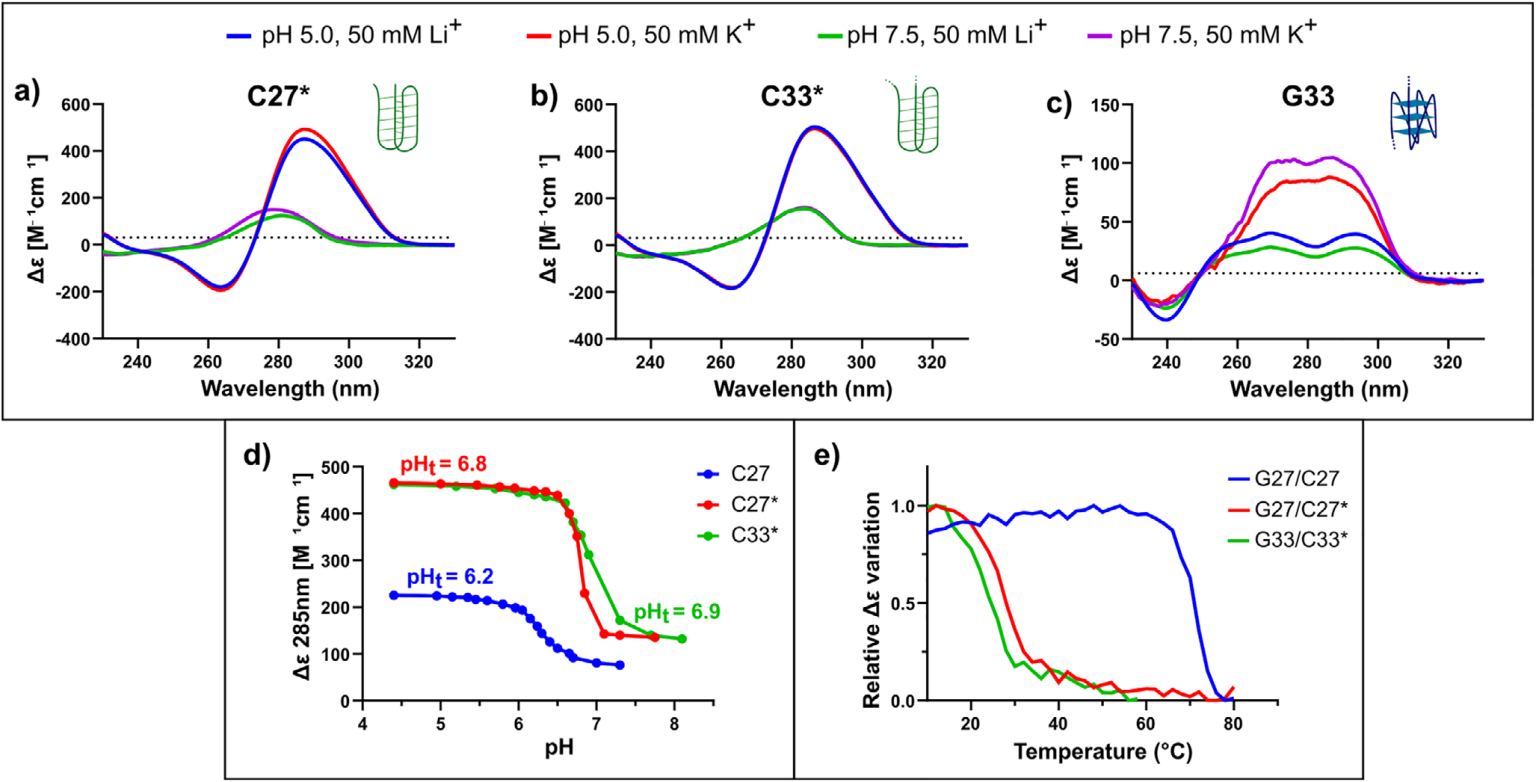
Optimization of the central core domain. (a–c) CD spectra acquired in cacodylate buffer at pH 7.0, with 50 mM KCl (purple lines) or 50 mM LiCl (green lines) and at pH 5.0, with 50 mM KCl (red lines) or 50 mM LiCl (blue lines) for 3 µM C27* (a), C33* (b), and G33* (c). (d) Variation of the CD signal recorded at 285 nm as a function of the pH for 3 µM C27 (blue line), C27* (red line) and C22* (green line) in cacodylate buffer, 50 mM KCl (e) Relative variation of the CD signal recorded at 270 nm as a function of the temperature for 30 µM G27/C27 (blue line), G27/C27* (red line), G33/C33* (green line) in cacodylate buffer, pH 5.0, 50 mM KCl.

However, physiological processes, such as transcription and replication, alter B‐DNA pairing and favor its transition toward noncanonical structures [54]. To mimic such a condition in our construct, we modified the C‐rich module to reduce its pairing to the G‐rich one while increasing its iM stability. We accomplished both goals by substituting C27, which folds into an iM with 6 C:C^+^ base pairs, with C27*, a sequence of the same length that was previously reported to fold into an iM with 9 C:C^+^ bp [8]. Consistently, when compared to C27 in acidic conditions, C27* showed a more intense CD signal at 285 nm (a parameter directly related to the number of C:C^+^ in the iM), and its pH transition (pH_t_) was shifted to higher values (6.8 vs. 6.2) (Figures 3a,d and S3) [55]. We also monitored the pairing of G27/C27*, which was predicted to form only 11 base pairs out of the 27 occurring in C27/G27. The CD melting/annealing profiles of these duplexes acquired at pH 7.5, 50 mM LiCl (to efficiently prevent both iM and G4), confirmed a drop of the *T*_m_ from 75°C for G27/C27 to 27°C for G27/C27* (Figures 3e and S4).

For C27*, a concern raised from the C:C^+^ pairing of the terminal C1 and C27 that might elicit the potentially useful flexibility provided by “hinge” domains at the interface between different structural modules in the full‐length construct. Thus, we elongated both terminals with a triplet (TTT for C27* and TTA for G27), obtaining C33* and G33. The longer C‐rich sequence preserved the iM‐folding of C27 with a similar pH of transition (pH_t_ 6.9; Figures 3b,d and S3). For G33, its chiroptical signal in 50 mM KCl slightly increased in comparison to G27. However, the preserved CD and TDS profiles pointed to their comparable G4 arrangement (Figures 3c and S5). Moreover, the thermal stability of the annealed G33/C33* (at pH 7.5, 50 mM LiCl) was only slightly reduced with reference to G27/C27* (T_m_ = 25°C vs. 27°C, Figures 3e and S4).

The CD melting profiles of these ds constructs were not acquired under conditions compatible with both iM and G4 formation (pH 5.0, 50 mM KCl) due to the expected coexistence of multiple arrangements at different temperatures. However, we applied them to derive the thermodynamic parameters of G33, C33*, Ff1, and Ff2 folding (Figure S6 and Table S2). They ranked the ds flanking domains as the most stable species, followed by iM, and finally, G4.

### 2.2 Secondary Structures Within the Partially Complementary Full‐Length Construct

**FIGURE 4:**
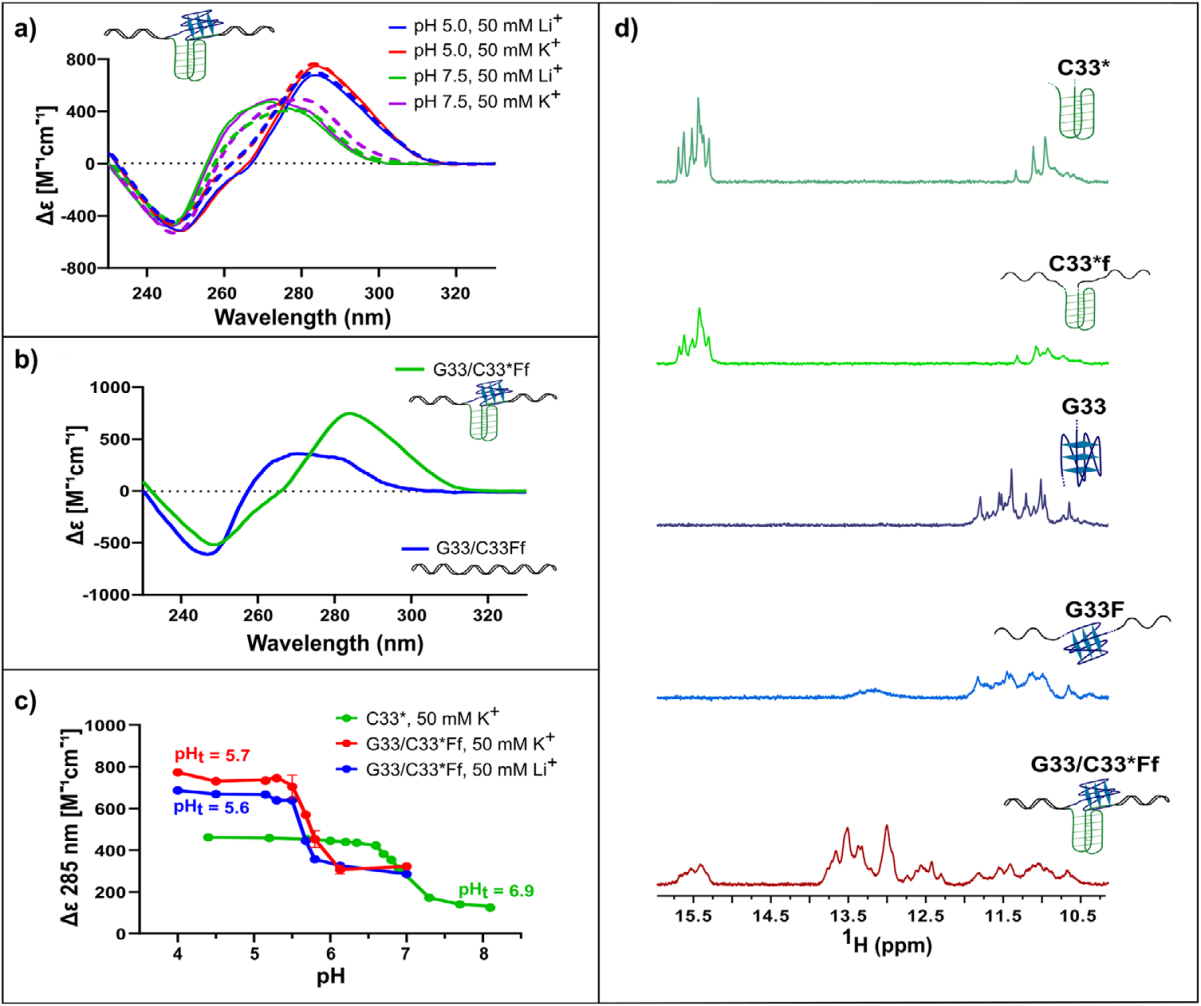
Folding of the G/C rich core within the partially complementary full‐length G33/C33*Ff construct. (a) Comparison of the experimentally acquired CD spectra (solid lines) with the linear combination of the reference spectra reported in Figure S1 (dotted lines) for 10 µM G33/C33*Ff in cacodylate buffer, pH 7.0, with 50 mM KCl (purple lines) or 50 mM LiCl (green lines) and at pH 5.0, with 50 mM KCl (red lines) or 50 mM LiCl (blue lines). (b) CD spectra of G33/C33*Ff (green line) and G33/C33Ff (blue line) acquired in cacodylate buffer, pH 5.0,50 mM KCl. (c) Variation of the CD signal as a function of the pH recorded at 285 nm in cacodylate buffer for 4 µM C33*, with 50 mM KCl (green line), 10 µM G33/C33*Ff, with 50 mM KCl (red line), and 10 µM G33/C33*Ff, with 50 mM LiCl (blue line). (d) Imino region of the ^1^H NMR spectra of 80 µM ss iM (C33*, C33*f) or G4 (G33, G33F) forming sequences and the full‐length G33/C33*Ff construct acquired in cacodylate buffer, pH 5.0, with 50 mM KCl, with 10% D_2_O.

Since G33/C33* was the most promising core unit to follow the occurrence of noncanonical structures within the full‐length construct, we characterized it under progressively more complex sequence environments. First, we compared the chiroptical signature of G33 and C33* when flanked by ss or ds domains. The good agreement between the predicted and the experimentally acquired CD spectra under all tested conditions confirmed that both iM‐ and G4‐folding were not affected by the presence of flanking domains (Figure S7). However, for the annealed full‐length G33/C33*Ff, the experimental data differ from the predicted ones (Figure 4a). In detail, at pH 7.0, the acquired spectra were independent of KCl, indicating a preserved canonical dsDNA arrangement (Figure 4b). Conversely, under acidic conditions, the positive signal at 285 nm indicated iM formation and, in KCl, the spectrum fitted the predicted one, thus suggesting the concomitant G4 presence. Indeed, although modest, the spectral variation observed moving from Li^+^ to K^+^ was reasonably compatible with G4‐folding since its positive CD signal is four‐fold lower than the iM one in the 270–290 nm range.

To gain deeper insights into the potential iM‐ and G4‐interplay within the central domain of G33/C33*Ff, we also performed pH titrations in cacodylate buffer with either 50 mM KCl or LiCl (Figure 4c). By following the CD signal at 285 nm, it emerged that within the full‐length construct, iM formation required more acidic conditions (pH_t_ 5.7 vs. 6.9, respectively). However, it was essentially independent of G4 occurrence (pH_t_ 5.7 or 5.6 in 50 mM KCl or LiCl, respectively), further supporting that they were not mutually exclusive. This model was in line with ^1^H NMR data (Figure 4d) acquired in cacodylate buffer, pH 5.0, with 50 mM KCl. Indeed, in the imino proton region, the signals associated with the ds flanking, iM and G4 were all detected in the full‐length G33/C33*Ff construct.

### 2.3 Validation of the Partially Complementary Full‐Length Construct by Selected Modifications

**FIGURE 5:**
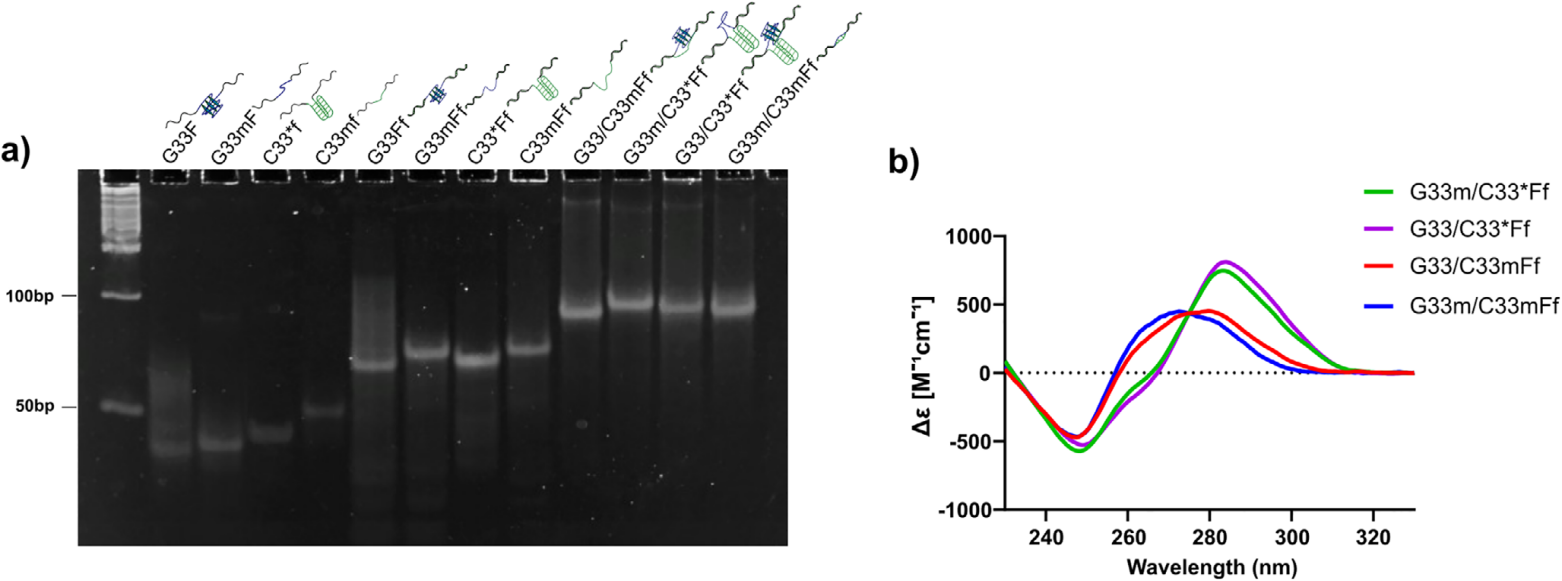
Characterization of modified constructs. (a) Native PAGE of ss (1.6 µM) and ds constructs (1 µM) in cacodylate buffer, pH 5.0, 50 mM KCl. (b) CD spectra of 10 µM G33/C33*Ff (violet line), G33m/C33*Ff (green line), G33/C33mFf (red line), and G33m/G33mFf (blue line) acquired in cacodylate buffer, pH 5.0, 50 mM KCl.

So far, we applied distinct experimental conditions, known to selectively modulate the folding propensity of individual structural domains, to probe their presence within the constructs under investigation. Nevertheless, these variations may simultaneously influence the thermodynamic profiles of multiple secondary structures, albeit to different extents. To overcome this bias, we introduced a few modifications in the central domain of G33F and C33*f to selectively prevent G4‐ or iM‐folding while keeping the same length and nucleotide composition. In detail, G33mF was derived by introducing two G to C modifications within the second and third G‐runs of G33F and C33m*f, by including the complementary C to G modifications on C33*f. CD spectra and TDS acquired in cacodylate buffer, pH 5.0, 50 mM KCl, confirmed a significant impairment of tetra‐helical structures formation by these modified sequences. Consistently, when analyzed by PAGE, these constructs displayed reduced electrophoretic mobility relative to the corresponding original (unmodified) wild‐type oligonucleotides (Figures S8, 5a). This behavior was fully retained when the flanking modules were in the ds form. Notably, each construct obtained from the annealing of different combinations of the original and modified full‐length strands (G33m/C33*Ff, G33/C33mFf, G33/C33*Ff, and G33/C33Ff) had distinctive electrophoretic mobilities and CD signatures, in perfect agreement with their expected different content in secondary structures (Figure 5b).

### 2.4 The Folding Pathway of The Partially Complementary Full‐Length Construct

**FIGURE 6:**
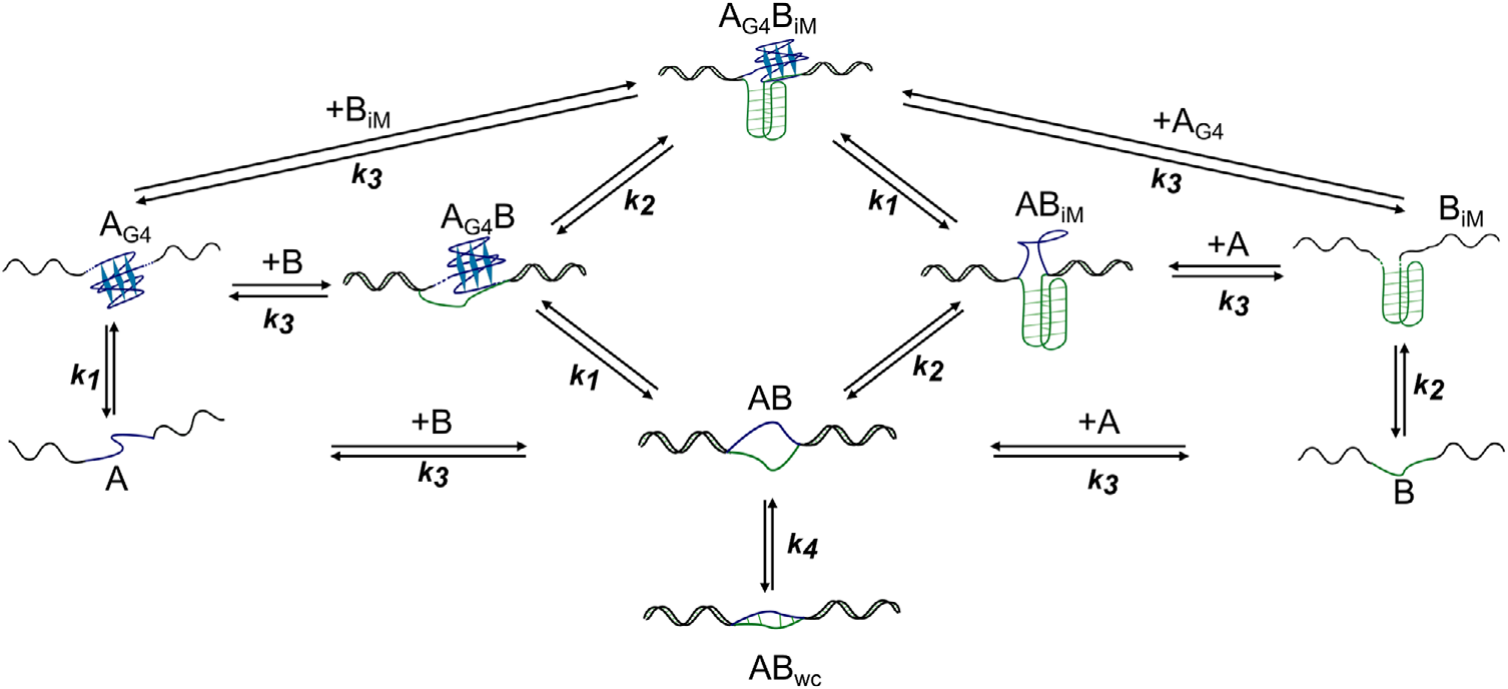
Model mechanism of transitions occurring within the partially complementary full‐length construct.

Overall, experimental evidence validated G33/C33*Ff as a suitable model construct to dissect the energetic contribution of ds‐, G4‐, and iM‐formation when clustered at a single site. At first, we acquired the CD melting/annealing profiles of the G33/C33*Ff in cacodylate buffer, pH 5.0, 50 mM KCl (Figure S9). The process was fully reversible and showed the two most relevant transitions. The first one, nicely followed at 248 nm, showed a *T*_m_ of about 49°C, which can be ascribed to the overlapping unfolding of the two flanking duplexes. Moving up to 285 nm, a second transition detected around 65°C can be ascribed to the iM unfolding. Unexpectedly, no clearly resolved process associated with G4 was detected, possibly due to its reduced chiroptical contribution with reference to iM and ds. However, DSC profiles acquired under the same conditions also showed two aligned transitions (Figure S9). Nevertheless, a closer look at the thermograms showed that they are asymmetric, which suggested that each of them reflects more than one transition. This finding led us to consider the more comprehensive thermodynamic model presented in Figure 6. It covers a relatively large (but relevant) number of equilibria and is thus inappropriate for a direct analytical description of the distribution of the different occurring species. As a reasonable approximation, we considered associating a conserved equilibrium constant of formation of each secondary structure irrespective of the surrounding sequence environment, that is, *k*_1_ and *k*_2_ to describe the formation of intramolecular G4 and iM, *k*_3_ for the formation of bimolecular flanking duplexes, and *k*_4_ for folding of the central partially paired domain. To validate whether the thermodynamic fingerprints of G4, iM, and ds were actually conserved across species at variable architectural complexity, we took advantage of the modular feature of our G33/C33*Ff construct as well as of the abovementioned related, modified sequences.

### 2.5 Thermodynamic Analysis of the Single‐Stranded Sequences

To derive the *k*_1_ and *k*_2_ associated with the (un)folding of G4 and iM, respectively, we followed the melting/annealing processes of C33*f and G33F by CD and DSC in cacodylate buffer, pH 5.0, 50 mM KCl at different oligonucleotide concentrations (1 and 250 µM, respectively) (Figures S10 and S11). Both techniques confirmed the reversibility of the process and identified the main transitions at comparable temperatures, in line with intramolecular folding. We fitted the CD profiles of G33F and C33*f (at 285 and 290 nm, respectively) with a two‐state model (Equation S1) and we derived the corresponding thermodynamic parameters (Table 1 and S3). For C33*f, they were in good agreement with those derived by fitting the symmetric DSC band with a two‐state model (Equation (S3) and Figure S10b) and with those above derived for the isolated iM domain C33* (Table S2). Unexpectedly, both DSC and UV melting at 260 nm of G33F highlighted two transitions (Figures S11a and S12a). TDS suggested the occurrence of canonical base pairing (a more intense signal at 260 nm for G33F in comparison to G33) while native PAGE showed a single band with high electrophoretic mobility (Figure S12b,c) within the entirely explored G33F concentration range (1–250 µM). Thus, we ascribed the transition occurring at a lower temperature (25°C) to an intramolecular pairing (hairpin). Since its formation is likely prevented when G33F is annealed to the partially complementary C33*f, we deconvoluted the DSC thermogram of G33F to isolate the transition at higher temperature, associated with G4‐folding, and we describe it according to Equation (S4) (Figure S10e, and Tables 1 and S3). The parameters derived by analysis of CD and DSC data are in good agreement and comparable to those estimated for the isolated G4 in G33 (Δ*G* −2.6 vs. −2.9 kcal mol^−1^, Table S2).

**TABLE 1:** Standard thermodynamic parameters of folding of G4, iM, and ds domains forming at full‐length ss (G33F and C33f) and ds (G33/C33mFf, G33m/C33*Ff, and G33m/C33mFf) constructs derived from the analyses of CD and/or DSC melting/annealing profiles acquired in cacodylate buffer, pH 5.0, 50 mM KCl.

Species	Sequence	Structural domain	Dataset	Δ*H* (kcal mol^−1^)	Δ*S* (cal mol^−1^ K^−1^)	Δ*G*_(298.15 K)_ (kcal mol^−1^)
*A*_G4_	G33F	G4	CD	−41.5 ± 2.1	−130.6 ± 2.2	−2.6 ± 2.3
	G33F	G4	DSC	−37.8 ± 0.6	−118.1 ± 1.8	−2.6 ± 0.1
*B*_iM_	C33*f	iM	CD	−79.5 ± 2.6	−233.6 ± 2.6	−7.3 ± 0.3
	C33*f	iM	DSC	−93.6 ± 1.7	−276.4 ± 5.3	−11.2 ± 0.2
*A*_G4_B	G33/C33mFf	ds	DSC	−121.7 ± 0.8	−350.5 ± 2.6	−17.2 ± 0.1
		G4	DSC	−35.0 ± 8.4	−113.9 ± 20.4	−2.9 ± 0.9
*AB*_iM_	G33m/C33*Ff	ds	DSC	−128.5 ± 3.7	−372.4 ± 11.6	−17.5 ± 0.3
		iM	DSC	−87.8 ± 3.0	−259.4 ± 8.5	−10.4 ± 0.4
*AB*_WC_	G33m/C33mFf	ds	DSC	−144.8 ± 3.9	−419.6 ± 12.3	−19.7 ± 0.3
		Central pairing	DSC	−76.4 ± 3.5	−236.4 ± 11.1	−6.0 ± 0.2

Overall, these results indicated that ss flanks do not significantly alter either iM‐ or G4‐folding.

### 2.6 Thermodynamic Analysis of the Modified ds Constructs

The previously characterized modified constructs turned out to be essential to address whether the derived *k*_1_ and *k*_2_ were preserved when G4 and iM formed within a ds environment. Indeed, the annealed G33m/C33*Ff and G33/C33mFf properly mimic *AB*_iM_ and *A*_G4_B, respectively. The G33m/C33mFf construct, corresponding to *AB*_WC_, was also included to derive *k*_4_. Their thermodynamic behaviors were followed by CD and DSC (10 and 30 µM, respectively) in cacodylate buffer, pH 5.0, 50 mM KCl (Figures S13 and S11). CD and DSC melting/annealing profiles were mutually consistent and suggested that the observed (un)folding transitions can be considered reversible. Due to the co‐presence of multiple species within every tested system, CD plots at specific wavelengths were used to properly address the species occurring along the main DSC transitions (Figure S14). Accordingly, for G33m/C33*Ff, the two resolved DSC transitions were associated with iM and ds flankings, as observed by plotting the CD signal as a function of temperature at 285 and 248 nm, respectively. In addition, the comparison of the CD melting profile at 285 nm of G33m/C33*Ff and G33/C33*Ff highlighted that a chiroptical contribution was lost around 50°C for the modified construct, in agreement with the absence of G4. Conversely, G33/C33mFf exhibited a single asymmetric DSC peak (Figure S11) at around 50°C, in agreement with the CD transitions measured at both 248 and 290 nm (Figure S13a,b). This output can refer to the overlapping of G4‐unfolding, characterized by a reduced enthalpic contribution, with the melting of the flanking duplexes. Consistently, the DSC thermogram of G33m/C33mFf indicated a broad transition in the same temperature range. Thus, although the isolated Ff1 and Ff2 duplexes showed somewhat different behavior (Table S2), we inferred that within the full‐length construct, their unfolding can be reasonably considered comparable, allowing us to describe both of them using a single constant (*k*_3_). Notably, only G33m/C33mFf displayed a clear shoulder at lower temperatures of the main DSC transition (Figure S13k), which we assigned to the opening of the partially paired central domain (*k*_4_). Based on these outputs, the thermograms of G33/C33mFf, G33m/C33*Ff, and G33m/C33mFf were fitted with the corresponding model functions described by Equations (S5–S7), respectively, providing the thermodynamic parameters and species distribution reported in Tables 1 and S3, and Figure S13. They confirmed that both G4 and iM retained their thermodynamic fingerprint when inserted within a DNA environment at different levels of molecular complexity (Δ*G*_1_ −2.9 ± 0.9 vs. −2.6 ± 2.3 kcal mol^−1^ for G33/C33mFf vs. G33F; Δ*G*_2_ −10.4 ± 0.4 vs. −11.2 ± 0.2 kcal mol^−1^ for G33m/C33*Ff vs. C33*f). Additionally, the derived Δ*H*_3_ associated with the ds domains (120−150 kcal/mol), is well aligned with the sum of the enthalpies determined for the isolated f Ff1 and Ff2 (∼135 kcal/mol, Table S2). The modest reduction of Δ*G*_3_ observed for both G33/C33mFf and G33m/C33*Ff (~−17.4 kcal mol^−^^1^) with reference to G33m/C33mFf (−19.7 kcal mol^−^^1^) may align with a possible destabilization/interaction of G4/iM with the adjacent duplexes [56, 57]. Last, the analysis of G33m/C33mFf allowed the quantitative characterization of the partially paired central domain (Δ*G*_4_ −6.0 ± 0.2 kcal mol^−1^).

### 2.7 Thermodynamic Analysis of the Partially Complementary Full‐Length Construct

**FIGURE 7:**
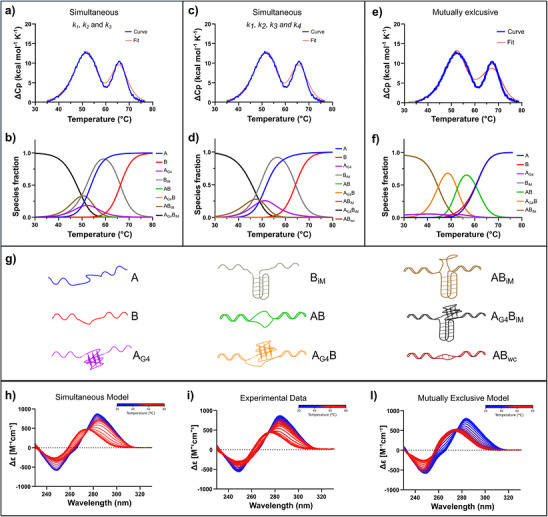
Validation of G33/C33*Ff thermodynamic model. (a–f) DSC thermogram of 30 µM G33/C33*Ff in cacodylate buffer, pH 5.0, 50 mM KCl fitted according to different G4‐iM formation models: simultaneous (a), Equations (S8), (c), (S9), or mutually exclusive (e), (S10). Blue and red lines correspond to experimentally acquired and fitting resulting curves, respectively. The corresponding derived species distribution plots are reported in (b), (d), and (f). Color lines correspond to the species drawn in (g–l) CD melting profile of G33/C33*Ff reconstructed from the parameters derived from the simultaneous (h) and mutually exclusive (l) model compared to the experimentally acquired dataset (i).

Having addressed that the equilibrium constants *k*_1_, *k*_2_, and *k*_3_ were reasonably conserved among the different species potentially occurring within our partially paired full‐length construct, we analyzed the DSC thermogram of G33/C33*Ff by two analytical models both of which account for the simultaneous presence of G4 and iM, while differing in whether the contribution of *k*_4_ was neglected (Equation S8) or explicitly included (Equation S9). Data analyses performed by including four equilibrium constants failed to detect any contribution associated with *AB*_WC_ (Table S4). To validate this output, in Equation (S9) we used as constraints the thermodynamic parameters previously determined for this species in the G33m/C33mFf model (Δ*H*_4_ = −80.0 kcal mol^−1^ and *T*_m4_ = 50°C). With this set‐up, both Equations (S8) and (S9) properly described the experimental data (Figure 7), and the provided thermodynamic parameters (Tables 2 and S5) were used to derive the corresponding species distributions (*R* ^2^ coefficient of 0.985 with both fitting equations). These plots further confirmed the contribution of *AB*_WC_ (dark red line in Figure 7d) as negligible, thus addressing Equation (S8) as reliable to properly describe our model. The obtained parameters were further compared to those above derived for the ss and ds constructs. While iM fingerprint was essentially conserved across the different constructs (Δ*G*_2_ −10.4 and −10.7 kcal mol^−1^, for G33/C33*Ff and G33/C33mFf, respectively, with comparable enthalpic and entropic contributions), the thermodynamic profile of G4 slightly changed (Δ*G*_1_ −3.8 and −2.9 kcal mol^−1^ for G33/C33*Ff and G33/C33mFf, respectively), mostly for an enthalpic difference (Δ*H*_1_ −54.3 and −35.0 kcal mol^−1^ for G33/C33*Ff andG33/C33mFf, respectively), consistent with the proposed G4 stabilization induced by the iM presence the complementary strand [37, 39]. Finally, the above‐reported destabilization of the flanking domains observed in the presence of either G4 or iM appeared to be further increased by their simultaneous occurrence, mostly as a result of a reduced enthalpic contribution (−107.3 vs. ~ −122–128 kcal mol^−1^) [57]. However, since in our experimental conditions G4 and ds transitions partly overlapped, a relative compensation along the analyses cannot be ruled out.

**TABLE 2:** Standard thermodynamic parameters of folding of G4, iM, and ds domains within the full‐length construct G33/C33*Ff derived by fitting of the DSC thermograms acquired in cacodylate buffer, pH 5.0, 50 mM KCl, according to Equations (S8), (S9), or (S10).

Equation	Domain	Δ*H* (kcal mol^−1^)	Δ*S* (cal mol^−1^ K^−1^)	Δ*G*_(298 K)_ (kcal mol^−1^)
(S8)	Duplex	−111.3 ± 1.2	−321.0 ± 3.5	−15.6 ± 0.1
	G4	−50.2 ± 6.5	−155.7 ± 20.1	−3.8 ± 0.5
	iM	−89.6 ± 2.8	−264.8 ± 7.6	−10.7 ± 0.6
(S9)^a^	Duplex	−107.3 ± 1.0	−308.7 ± 2.8	−15.2 ± 0.1
	G4	−54.3 ± 3.8	−168.3 ± 11.8	−4.1 ± 0.3
	iM	−89.4 ± 3.0	−264.1 ± 7.9	−10.7 ± 0.6
(S10)	Duplex	−109.8 ± 1.8	−305.0 ± 6.0	−18.8 ± 0.1
	G4	−91.1 ± 4.8	−279.6 ± 15.1	−7.7 ± 0.3
	iM	−162.7 ± 7.3	−504.6 ± 12.2	−12.2 ± 0.4

^a^ Calculated using Δ*H*_4_ = −80.0 kcal mol^−1^ and *T*_m4_ = 50°C as constrains.

In line with the overlapping CD spectra of G33/C33*Ff at pH 7.0, with K^+^ or Li^+^ (Figure 4a), both models confirmed that within our construct, G4 was absent when the complementary iM was unfolded (see A_G4_B distribution (orange line) in Figure 7), while iM was conserved also upon G4‐unfolding (*AB*_iM_ species (brown line)). This result is quantitatively described by the sequence of absolute values Δ*G*_2_ > Δ*G*_4_ > Δ*G*_1_: it illustrates how iM formation efficiently drives the opening of the partially paired central domain, eliciting its competition versus G4‐folding. This observation is in line with the model of Sun and coworkers, where [39], under neutral conditions, iM was required to support G4‐folding at the complementary site. However, although they consider a possible lag‐time between the iM‐unfolding and its resolution of the G4, when this occurred, the full complementarity of the two strands made the process irreversible. Conversely, our data quantitatively demonstrated how the relative thermodynamic stabilities of the different structural domains effectively tune the structural transitions within duplex DNA under reversible conditions.

As a further validation step, we also analyzed the DSC profiles according to a mutually exclusive G4 and iM model that included *k*_1_, *k*_2_, and *k*_3_ (Equation S10) (Figure 7e,f). Although it did not optimally describe the DSC profile of G33/C33*Ff, we used it to derive the thermodynamic parameters and the species distribution (Table 2, S5). It emerged that this model overestimated the absolute Δ*G* values associated with each species with reference to those obtained from ss and modified constructs. Moreover, we used our experimentally acquired CD spectra corresponding to all the species involved to reconstruct the CD melting profile of G33/C33*Ff from the species distribution derived according to both the simultaneous and mutually exclusive models (Equation S11). As reported in Figure 7g–i, the comparison of the experimental and predicted CD melting data set (Figure S9a), further demonstrated that the simultaneous model provided a more reliable description of G33/C33*Ff melting profile compared to the mutually exclusive one. The plot at 285 nm of distinct data sets further confirmed the good description of the system according to the simultaneous model (Figure S15).

Overall, this analysis provided a consistent description of the G4 and iM thermodynamic properties and distribution when colocalized within a genomic environment. Notably, it showed that while iM was detected even when G4 was unfolded, the reverse did not occur (see the distribution of *AB*_iM_ (brown line) and A_G4_B (orange line) in Figure 7). This prompted us to test the robustness of our model under slightly less acidic conditions, which should impact mostly on iM stability. The DSC thermogram of G33/C33*Ff acquired at pH 5.2 (Figure S16a) showed a reduced resolution of the two main contributions. As supported by its description with Equation S8, this output was mostly related to a reduced stability of the iM‐containing species (Figure S16 and Table S6). Indeed, the unfolding of both G4 and lateral ds occurred at comparable temperatures to those observed at pH 5.0, thus confirming the flanking duplexes as the most stable domains across both tested conditions. Conversely, for iM, a larger enthalpic contribution at less acidic pH compensated for the unfavorable entropic term, resulting in a conserved Δ*G*_2_ within this pH range. This behavior is related to the differences in oligonucleotide protonation obtained by changing the pH: while at pH closer to cytosine *pK*a (4.6), a higher number of cytosines are protonated, at increasing pH, an additional enthalpic contribution is required to protonate the (still) unprotonated C:C^+^ base pairs to allow iM formation [58]. Nevertheless, iM energetic variations within the full‐length construct were sufficient to modify the relative species distribution. Notably, the A_G4_B_iM_ species, corresponding to the simultaneous presence of G4 and iM embedded within the ds flanking, is preserved at low temperatures. This output confirmed our thermodynamic model as a suitable tool to describe the species distribution perturbation in response to environmental changes.

### 2.8 Small‐Angle X‐Ray Scattering

**FIGURE 8:**
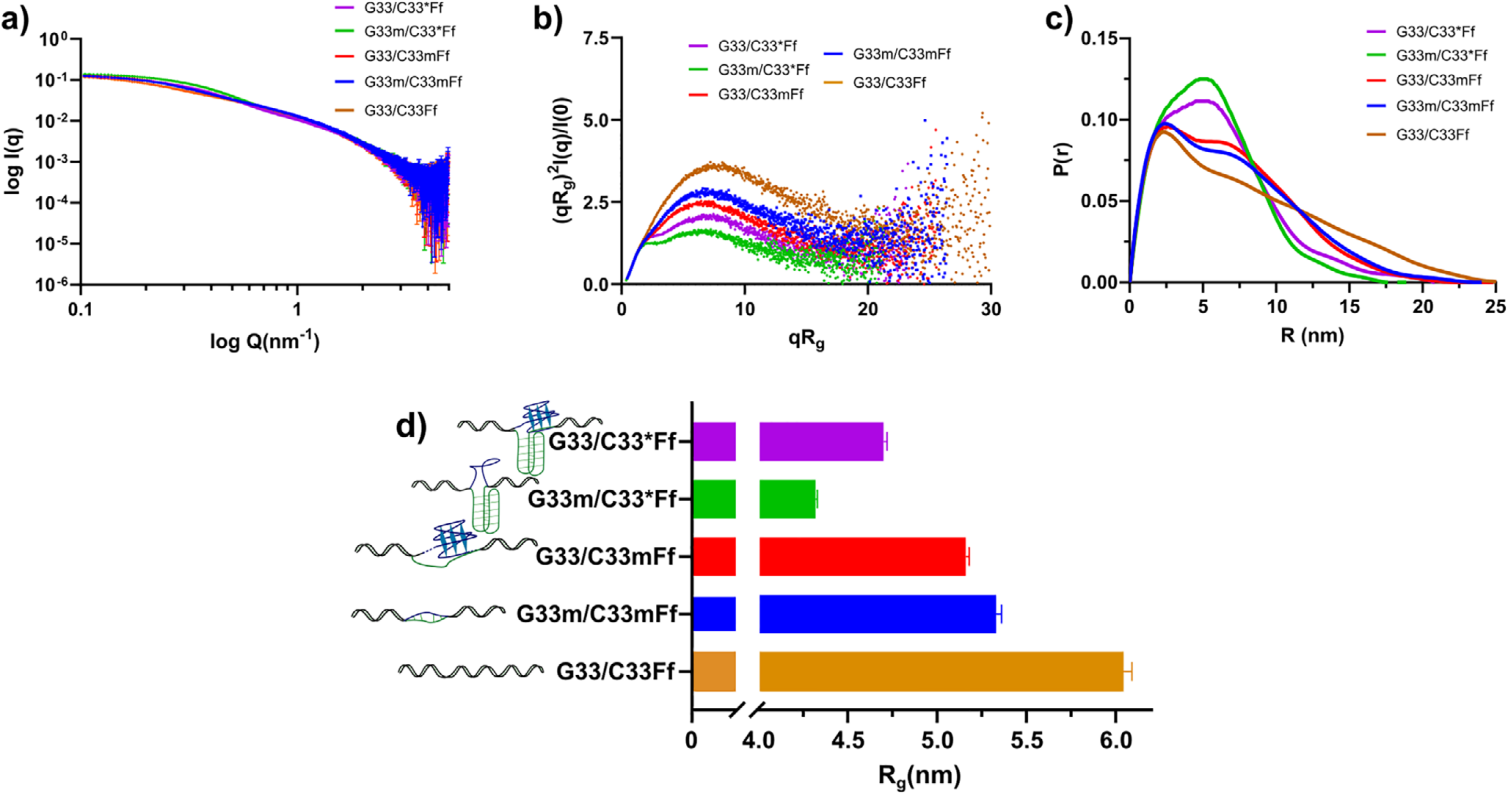
SAXS characterization of full‐length constructs. (a) Intensity data, (b) Krakty Plot, (c) P(r) profile, and (d) *R*_g_ values derived from Guiner's region of 25 µM G33/C33*Ff (violet line), G33m/C33*Ff (green line), G33/C33mFf (red line), G33m/C33mFf (blue line) and G33/G33Ff (orange line), acquired in cacodylate buffer, pH 5.0, 50 mM KCl.

The observed variation in iM and G4 occurrence within the full‐length constructs can be reasonably related to alterations in the shape, size, and relative geometry of the constituent structural domains. To address this point in solution, we performed SAXS measurements of G4 and iM, both as singular domains and embedded within lateral ss and ds flankings in cacodylate buffer, pH 5.0, 50 mM KCl. Intensity profiles (Figures 8a and S17) were analyzed using a model‐independent approach to determine structural parameters, including the maximum particle dimension (*D*_max_) and the radius of gyration (*R*_g_), which describes the spatial distribution of electron density around the center of mass and is a key parameter for characterizing the overall size and conformational changes of macromolecules in solution. In the case of our samples, which consist of multiple modules within a single object, it reflects the average spatial distribution of the tested assembly in solution (Table S7). For all our tested constructs, these two parameters were consistent with each other.

As a preliminary analysis, we compared G33 to the shorter telomeric sequence G22, which has already been studied by SAXS [59]. The *R*_g_ for G22 was in line with the reported hybrid telomeric G4. The longer G33 showed an increment of *R*_g_ from 1.19 to 1.58 nm and an enhanced flexibility, as shown by the dimensionless Kratky plots where the two sequences share a defined maximum of 1.104 at *qR*_g_ = 1.73(√3), consistent with a folded G4 structure, whereas the longer one reached a plateau at higher *qR*_g_ (Figure S17d) [60], suggesting the presence of a more flexible structure. This output was also supported by the electron pair‐distance distribution function P(r): while a symmetric, bell‐shaped curve was observed for G22, consistent with a globular and compact G4 (Figure S17g), a wider, asymmetric P(r) profile was obtained with G33. Notably, the parameters and structural features for C33* folded into an iM (R_g_ of 1.52 nm) were comparable to those of G33. As expected, the longer ss sequences G33F and C33*f (Figure S17b,e,h) provided comparable R_g_ (3.34 and 3.40 nm), despite a marked difference in the P(r) profiles, suggesting a different relative orientation of the ss flanking with respect to the G4 and iM cores. Indeed, in the absence of non‐canonical folding, the G33mF and C33mf constructs exhibit larger *R*_g_ of 4.1 and 4.0 nm, respectively. The dimensionless Kratky plots of G33mF and C33mf are consistent with the presence of completely unfolded strands (Figure S17e). However, from the SAXS intensity curves and the P(r) distributions, a partially folded state can be inferred for C33mF, as supported also by TDS and CD data (Figures S17b,h and S8). When the flanking regions were in the ds form, the SAXS‐derived parameters were in line with the electrophoretic profiles. Indeed, the modified constructs showed reduced *R*_g_ values when compared to the corresponding sequences properly folded into the noncanonical structures (Table S7).

The most relevant outcome derived from comparing the full‐length constructs (Figure 8). As expected, the fully paired G33/C33Ff was the most elongated with a rod‐like P(r) profile consistent with a canonical double helix [61]. Partial pairing in the modified G33m/C33mFf decreased duplex rigidity, yielding a more compact conformation (reduced *R*_g_ and a less pronounced rod‐like character in P(r) distribution). This feature was fully retained by modified constructs tested under experimental conditions that prevented noncanonical arrangements (Table S8). Only at pH 7.5, *R*_g_ slightly increased, likely due to more efficient pairing of the two partially complementary strands when cytosines were not protonated.

The G4 folding in this domain (G33/C33mFf) resulted in a modest decrease in *R*_g_, whereas iM formation (G33m/C33*Ff) produced a substantial reduction. This output might be unexpected since both G4 and iM structures generate more compact forms than the corresponding fully or partially paired ds central domain. However, based on our sequence design, the number of bases involved in G4 formation was reduced with reference to those paired in iM, and thus it leaves a larger fraction of the sequence unpaired and flexible. As a result, even though the G4 core itself is more compact than the iM, the overall *R*_g_ can be larger because the scattering centers are, on average, distributed farther from the center of mass. Notably, when both the iM and G4 structures can be accommodated (G33/C33*Ff), the structural parameters and SAXS profiles converged toward those measured for constructs in which only one of the two motifs can fold. Specifically, for G33/C33*Ff, the P(r) distribution was similar to that of G33m/C33*Ff, while the *R*_g_ more closely matched that of G33/C33mFf, supporting the coexistence of the two non‐canonical structures. Moreover, for all these partially paired constructs, the associated P(r) profiles are best fitted by multidomain models (Figure 8c).

## 3 Conclusion

In agreement with recent evidence, our data confirmed that G4 and iM can be simultaneously present at the two opposite strands of a DNA double helix, thus further ruling out steric hindrance as the only cause responsible for their mutual exclusivity. In addition, for the first time, here we succeeded in providing a thermodynamic model to describe the rationale behind this output. As expected, along this process, the energetic penalty associated with the local opening of GC‐rich regions must be compensated by the formation of noncanonical tetra‐helical structures. Such a barrier can be reduced during common biological processes (i.e., supercoiling, replisome, and protein interaction). Under these conditions, which we mimicked by reducing the pairing of the GC‐rich domain in our tested construct, we confirmed that G4 and/or iM folding can be more thermodynamically favorable over the topologically perturbed ds pairing.

The use of a modular construct allowed us not only to progressively optimize each individual segment to reach this energetic balance but also to compare its folding profiles to those determined when inserted within different structural environments. Notably, this analysis highlighted that the thermodynamic signatures of isolated G4 and iM structures were largely preserved when occurring within a ds environment, with only a slight G4 stabilization induced by iM folding on the opposite strand. This outcome foresees the possibility of predicting the distribution of secondary structures within a dsDNA fragment simply from the knowledge of the thermodynamic parameters of the isolated components.

However, it is worth noting that the present work focused only on a single GC‐rich pair. Although the selected G‐rich telomeric sequence can fold into diverse G4 topologies, the behavior of G4 and iM modules with different constrained topologies, as well as the impact of their possible structural rearrangements when folding within a longer DNA fragment, needs to be considered. Indeed, they can influence not only the thermodynamic profiles but also the final relative spatial orientation of coexisting noncanonical motifs, ultimately defining the G4–iM crosstalk. This clearly emerged from our model‐independent analysis of SAXS data, which showed how combined G4–iM folding within the duplex DNA leads to a multidomain architecture more compact than the canonical double helix. Notably, the folding of G4, iM, or both appeared to modulate the construct shape to a different extent, with iM leading to the most compact species. Combining the SAXS analyses presented here with appropriate computational approaches could provide further insights for refining current structural models [56, 62, 63].

Overall, our results confirmed this working system as a flexible platform to study the structural and thermodynamic interplay of diverse G4 or iM structures (and their variable combination) when inserted within a ds environment. Thus, it can be proficiently exploited to characterize the folding of non‐canonical structures with distinct topologies (i.e., parallel and antiparallel G4s, 3′‐ and 5′‐endo iMs) and under different experimental conditions, providing in the future a more rigorous evaluation of their potential cooperation in the fine‐tuning of biological mechanisms.

## Author Contributions

**Davide Auricchio**: investigation, methodology, writing – original draft, writing – review and editing, formal analysis, visualization, software. **Michele Ghezzo**: methodology, supervision, software, formal analysis, conceptualization. **Uroš Zavrtanik**: data curation, supervision, formal analysis, writing – review and editing, validation. **Luca Bertini**: writing – review and editing, methodology, supervision, formal analysis, validation. **Valeria Libera**: supervision, formal analysis, writing – review and editing, methodology, resources. **Riccardo Rigo**: methodology, formal analysis, investigation. **Jurij Lah**: data curation, supervision, validation, formal analysis, writing – review and editing. **Claudia Sissi**: conceptualization, funding acquisition, writing – original draft, writing – review and editing, visualization, project administration, data curation, supervision, resources, methodology, validation.

## Conflicts of Interest

The authors declare no conflicts of interest.

## Supporting information




**Supporting File**: The authors have cited additional references within the Supporting Information [64–67].

## Data Availability

The data that support the findings of this study are available from the corresponding author upon reasonable request.
