# Supplementary material for: Unraveling G‐Quadruplex and i‐Motif Coexistence Within a Double‐Stranded DNA
Source: Angew Chem Int Ed Engl. 2026 Jun 1;65(31):e3958607. doi: 10.1002/anie.3958607 (PMC13411504; doi:10.1002/anie.3958607)
Supplement: Supplementary file 1 — Supporting File: The authors have cited additional references within the Supporting Information [64–67]. [file ANIE-65-e3958607-s001.docx]

**Supporting Information**

Unraveling G-Quadruplex and i-Motif Coexistence Within a Double Stranded DNA

Davide Auricchio^[a]^, Michele Ghezzo^[a]^, Uroš Zavrtanik^[c]^, Luca Bertini^[b]^, Valeria Libera^[b]^, Riccardo Rigo^[a]^, Jurij Lah^[c]*^, Claudia Sissi*^[a]^

[a] Dr. Davide Auricchio, Dr. Michele Ghezzo, Dr. Riccardo Rigo, Prof. Claudia Sissi
Department of Pharmaceutical and Pharmacological Sciences,
University of Padova
Via Marzolo 5, 35131 Padova, Italy.
E-mail: [claudia.sissi@unipd.it](mailto:claudia.sissi@unipd.it)

[b] Dr. Luca Bertini, Dr. Valeria Libera,
Department of Physics and Geology,
University of Perugia,
via Alessandro Pascoli, 06123 Perugia, Italy.

[c] Dr. Uroš Zavrtanik, Prof. Jurij Lah
Department of Physical Chemistry, Faculty of Chemistry and Chemical Technology,
University of Ljubljana,
Večna pot 113, Ljubljana, Slovenia.
E-mail: [jurij.lah@fkkt.uni-lj.si](mailto:jurij.lah@fkkt.uni-lj.si)

**Table of Contents**

MATERIAL AND METHODS……………………………………………………………………………………………3

OLIGONUCLEOTIDES………………………………………………………………………………………..3

CIRCULAR DICHROISM (CD).……………………………………………………………………………….3

DIFFERENTIAL SCANNING CALORIMETRY (DSC)……………………………………………………...4

DSC DATA ANALYSIS…………………………………………………………………………………………4

RECONSTRUCTION OF CD MELTING PROFILE OF G33/C33*Ff FROM DSC-DERIVED SPECIES DISTRIBUTION…………………………………………………………………………….……………………………4

NATIVE GEL ELECTROPHORESIS.………………………………………………………………………..7

TDS……………………………………………………………………………………………………………...7

NMR……………………………………………… …………………………………………………………...7

SAXS…………………………………………………………………………………………………………….7

SUPPLEMENTARY FIGURES…………………………………………………………………………………………8

SUPPLEMENTARY TABLES………….………………………………………………………………………………19

REFERENCES………….……………………………………………………………………………………………...22

**Material and Methods**

**Oligonucleotides**

The sequences of the oligonucleotides used in this study are reported in Table S1. They were purchased from Eurogentec (Liège, Belgium). Stock solutions at 1 mM (strand concentration) were prepared by dissolving the lyophilized samples in H_2_O. For all the experiments, they were diluted to the required concentration in cacodylate buffer (50 mM lithium cacodylate) at the required pH, with 50 mM LiCl or 50 mM KCl. Before use, each sample was heated at 95°C for 5 min and then slowly cooled down to room temperature to equilibrate the system. For double-stranded constructs, the annealing was performed on samples containing an equimolar mixture of the two strands in the required buffer.

**Circular Dichroism**

CD spectra were acquired using a Jasco J-815 spectropolarimeter equipped with a Peltier accessory for temperature control, using a quartz cuvette with either 0.1 cm or 1 cm path length. Oligonucleotide samples were prepared in cacodylate buffer, adjusted to the appropriate pH and salt conditions, and analyzed within the 1-30 μM strand concentration. CD spectra were recorded from 230 to 330 nm. Each spectrum represents the average of 2 scans acquired with the following parameters: scanning speed 100 nm/min; band width of 2 nm; data interval of 0.5 nm; response of 2 s. For CD melting experiments, spectra were acquired between 20 and 80 °C with a step of 2 °C.

After subtraction of the buffer background, each CD spectrum was normalized by strand concentration using the following formula: Δε=θ*M/(C*L*32980), in units [M^−1^cm^−1^], where θ is the value expressed in millidegrees, M is molecular weight (g/mol), C is the g/L concentration, L is the path length of the cell.

The predicted CD spectra of the constructs were obtained by performing the algebraic sum of the CD spectra of each individual domains (G(x) C(y) F1, F2, f1, f2) included in the construct under investigation: experimentally acquired under the required experimental conditions:

ExpCD_G(X)F_ = CD_G(X)_ + CD_F1_ +CD_F2_

ExpCD_C(Y)f_ = CD_C(Y)_ + CD_f1_ +CD_f2_

ExpCD_G(X)Ff_ = CD_G(X)_ + CD_Ff1_ + CD_Ff2_

ExpCD_C(Y)Ff_ = CD_C(Y)_ + CD_Ff1_ + CD_Ff2_

ExpCD_G(X)/C(Y)Ff_ = CD_G(X)_ + CD_C(Y)_+ CD_Ff1_ + CD_Ff2._

CD Melting and annealing experiments were carried out in the 20°-90°C range at a 0.33 °C/min scan rate. Melting and annealing curves were corrected by signal drift by applying a linear regression of the upper and lower temperature data points according to the protocol described by J.L. Mergny and L. Lacroix^[62]^.

The fitting of the melting and annealing curves was performed according to equation 1 (concentration-independent equilibrium) for monomolecular species, such as G4 or iM and according to equation 2 (concentration-dependent equilibrium) for bimolecular ones:

$\Delta\varepsilon= \frac{a+b {e^{-}}^{\frac{\Delta Gꝋ\left( T^{\circ} \right)}{R T^{\circ}}-\frac{\Delta Hꝋ}{R}(\frac{1}{T}-\frac{1}{T^{\circ}})}}{1+{e^{-}}^{\frac{\Delta Gꝋ\left( T^{\circ} \right)}{R T^{\circ}}-\frac{\Delta Hꝋ}{R}(\frac{1}{T}-\frac{1}{T^{\circ}})}}$ (1)

$\Delta\varepsilon=a+ \frac{b-a}{1+e^{-ln([{B]}_{0})+\frac{\Delta Gꝋ\left( T^{\circ} \right)}{\mathrm{RT}}}}$ (2)

where T is the absolute temperature ( in K), T° is 298.15 K (25 °C), ΔG_ꝋ_(T°) is the standard Gibbs free energy change of folding (kcal mol^-1^) referred to T°, ΔH_ꝋ_ is the standard enthalpy of folding (kcal mol^-1^), R is the ideal gas constant (1.987 10^-3^ kcal mol^-1^ K^-1^), [B]_0_ is the oligo concentration, a and b are the Δε (M^-1^cm^-1^) of the unfolded and folded species, respectively. Data analysis and fitting were performed using NumPy and SciPy Python libraries. Parameter errors represent standard deviations obtained from the fitting procedure as the square roots of the diagonal elements of the corresponding variance-covariance matrix.

**Differential Scanning Calorimetry (DSC)**

DSC experiments were performed using Nano DSC instrument (TA Instruments, New Castle, DE, U.S.). Oligonucleotide samples were prepared in cacodylate buffer, adjusted to a pH within the 5.0-5.2 range, with 50 mM KCl. For these studies, samples were prepared at 250 µM, 150 µM, and 30 µM strand concentration for G33F, C33*f, and double-stranded constructs, respectively. Cyclic melting and cooling scans were performed in the 1°-90°C range at a 0.33 °C/min scan rate. For each measurement, baselines were acquired by filling both sample and reference cells with the required buffer. Baseline scans were subtracted from the melting/cooling scans, and data were reported as molar excess heat capacity (ΔC_p_(kcal/mol*K)).

**DSC data analysis**

Thermograms of each sample were fitted with model functions described by the equations reported here.

- **C33*f**

1. B *^k^_2_* B_iM_ $k2=\frac{[BiM]}{[B]}$

$Cp=\Delta H2 \frac{d(BiM)}{d(T)}$ (3)

where ΔH_2_ refers to the enthalpy change accompanying the iM unfolding process -transition of species B_iM_ to B (*k_2_*);

- **G33F**

1. A *^k^_1_* A_G4_ $k1=\frac{[AG4]}{[A]}$

$Cp=\Delta H1 \frac{d(AG4)}{d(T)}$ (4)

where ΔH_1_ refers to the enthalpy change accompanying the G4 unfolding process - transition of species A_G4_ to A (*k_1_*);

- **G33/C33mFf**

1. A *^k^_1_* A_G4_ $k1= \frac{[AG4]}{[A]}$
2. AB *^k^_1_* A_G4_B $k1= \frac{[AG4B]}{[AB]}$
3. A + B *^k^_3_* AB $k3=\frac{[AB]}{\left[ A \right]*[B]}$
4. A_G4_ + B *^k^_3_* A_G4_B $k3= \frac{[AG4B]}{\left[ AG4 \right]*[B]}$

$Cp=\Delta H1 \frac{d(AG4)}{d(T)}$ + $\Delta H3 \frac{d(AB)}{d(T)}+(\Delta H1$+ $\Delta H3$) $\frac{d(AG4B)}{d(T)}$ (5)

where ΔH_1_ refers to the enthalpy change accompanying the G4 unfolding process -transitions of species A_G4_ to A, and A_G4_B to AB (*k_1_*); ΔH_3_ refers to the enthalpy change accompanying unfolding of the lateral duplexes – transitions from AB to A and B and A_G4_B to A_G4_ and B (*k_3_*);

- **G33m/C33*Ff**

1. B *^k^_2_* B_iM_ $k2= \frac{[Bf]}{[B]}$
2. AB *^k^_2_* AB_iM_ $k2= \frac{[ABiM]}{[AB]}$
3. A + B *^k^_3_* AB $k3=\frac{[AB]}{\left[ A \right]*[B]}$

1. A + B_iM_ *^k^_3_*  AB_iM_ $k3= \frac{[ ABiM]}{\left[ A \right]*\left[ BiM \right]}$

$Cp=\Delta H2 \frac{d(BiM)}{d(T)}$ + $\Delta H3 \frac{d(AB)}{d(T)}+(\Delta H2$+ $\Delta H3$) $\frac{d(ABiM)}{d(T)}$ (6)

where ΔH_2_ refers to the enthalpy change accompanying the iM unfolding process - transitions of species B_iM_ to B and AB_iM_ to AB (*k_2_*); ΔH_3_ refers to the enthalpy change accompanying unfolding of the lateral duplexes – transition of species AB to A and B, and species AB_iM_ to A and B_iM_ *(k_3_*);

- **G33m/C33mFf**

1. A + B *^k^_3_* AB $k3=\frac{[AB]}{\left[ A \right]*[B]}$
2. AB *^k^_4_* AB_wc_ $k4=\frac{[ABwc]}{\left[ AB \right]}$

$Cp=$ $\Delta H3\frac{d(AB)}{d(T)}+ (\Delta H3$+ $\Delta H4$) $\frac{d(ABwc)}{d(T)}$ (7)

where ΔH_4_ refers to the enthalpy change accompanying the monomolecular unfolding process of the central domain in duplex form – transition of species AB_wc_ to AB (*k_4_*)^[63]^; ΔH_3_ refers to the enthalpy change accompanying the unfolding of lateral duplexes – transition of species AB to A and B (*k_3_*);

- **G33/C33*Ff: simultaneous formation model (3 constants)**

1. A *^k^_1_* A_G4_ $k1= \frac{[AG4]}{[A]}$
2. AB *^k^_1_* A_G4_B $k1= \frac{[AG4B]}{[AB]}$
3. AB_iM_  *^k^_1_* A_G4_B_iM_ $k1= \frac{[AG4BiM]}{[ABiM]}$
4. B *^k^_2_* B_iM_ $k2= \frac{[BiM]}{[B]}$
5. AB *^k^_2_* AB_iM_ $k2= \frac{[ABiM]}{[AB]}$
6. A_G4_B *^k^_2_* A_G4_B_iM_ $k2= \frac{[AG4BiM]}{[AG4B]}$
7. A + B *^k^_3_* AB $k3=\frac{[AB]}{\left[ A \right]*[B]}$
8. A_G4_ + B *^k^_3_* A_G4_B $k3= \frac{[AG4B]}{\left[ AG4 \right]*[B]}$
9. A + B_iM_ *^k^_3_* AB_iM_ $k3= \frac{[ ABiM]}{\left[ A \right]*\left[ BiM \right]}$
10. A_G4_ + B_iM_ *^k^_3_* A_G4_B_iM_ $k3= \frac{[ AG4BiM]}{\left[ AG4 \right]*[BiM]}$

$Cp=\Delta H1\frac{d\left( AG4 \right)}{d\left( T \right)}+\Delta H2\frac{d\left( BiM \right)}{d\left( T \right)}$+$\Delta H3\frac{d\left( \mathrm{AB} \right)}{d\left( T \right)}+(\Delta H1$+$\Delta H3$) $\frac{d\left( AG4B \right)}{d\left( T \right)}+(\Delta H2$+ $\Delta H3$)$\frac{d\left( \mathrm{AB}iM \right)}{d\left( T \right)}+(\Delta H1+\Delta H2\text{+}\Delta H3\text{)}\frac{d(AG4BiM)}{d(T)}$ (8)

where ΔH_1_ refers to the enthalpy change accompanying the G4 unfolding process - transitions of the species A_G4_ to A, A_G4_B to AB, and A_G4_B_iM_ to AB_iM_ (*k_1_*); ΔH_2_ refers to the enthalpy change accompanying the iM unfolding process - transitions of the species B_iM_ to B, AB_iM_ to AB, A_G4_B_iM_ to A_G4_B; ΔH_3_ refers to the enthalpy change accompanying the unfolding of lateral duplexes – transition of species AB to A and B, A_G4_B to A_G4_ and B, AB_iM_ to A and B_iM_, and A_G4_B_iM_ to A_G4_ and B_iM_.(*k_3_*);

**G33/C33*Ff: simultaneous formation model (4 constants**)

1. A *^k^_1_* A_G4_ $k1= \frac{[AG4]}{[A]}$
2. AB *^k^_1_* A_G4_B $k1= \frac{[AG4B]}{[AB]}$
3. AB_iM_  *^k^_1_* A_G4_B_iM_ $k1= \frac{[AG4BiM]}{[ABiM]}$
4. B *^k^_2_* B_iM_ $k2= \frac{[BiM]}{[B]}$
5. AB *^k^_2_* AB_iM_ $k2= \frac{[ABiM]}{[AB]}$
6. A_G4_B *^k^_2_* A_G4_B_iM_ $k2= \frac{[AG4BiM]}{[AG4B]}$
7. A + B *^k^_3_* AB $k3=\frac{[AB]}{\left[ A \right]*[B]}$
8. A_G4_ + B *^k^_3_* A_G4_B $k3= \frac{[AG4B]}{\left[ AG4 \right]*[B]}$
9. A + B_iM_ *^k^_3_* AB_iM_ $k3= \frac{[ ABiM]}{\left[ A \right]*\left[ BiM \right]}$
10. A_G4_ + B_iM_ *^k^_3_* A_G4_B_iM_ $k3= \frac{[ AG4BiM]}{\left[ AG4 \right]*[BiM]}$
11. AB *^k^_4_* AB_wc_ $k3=\frac{[ABwc]}{\left[ AB \right]}$

$Cp=\Delta H1\frac{d\left( AG4 \right)}{d\left( T \right)}+\Delta H2\frac{d\left( BiM \right)}{d\left( T \right)}$+$\Delta H3\frac{d\left( \mathrm{AB} \right)}{d\left( T \right)}+(\Delta H1$+$\Delta H3$)$\frac{d\left( AG4B \right)}{d\left( T \right)}+(\Delta H2$+$\Delta H3$)$\frac{d\left( \mathrm{AB}iM \right)}{d\left( T \right)}+(\Delta H1+\Delta H2\text{+}\Delta H3\text{)}\frac{d(AG4BiM)}{d(T)}$ $+ (\Delta H3$+$\Delta H4$)$\frac{d(ABwc)}{d(T)}$ (9)

where ΔH_1_ refers to the enthalpy change accompanying the G4 unfolding process - transitions of the species A_G4_ to A, A_G4_B to AB, and A_G4_B_iM_ to AB_iM_ (*k_1_*); ΔH_2_ refers to the enthalpy change accompanying the iM unfolding process - transitions of the species B_iM_ to B, AB_iM_ to AB, A_G4_B_iM_ to A_G4_B; ΔH_3_ refers to the enthalpy change accompanying the unfolding of lateral duplexes – transition of species AB to A and B, A_G4_B to A_G4_ and B, AB_iM_ to A and B_iM_, and A_G4_B_iM_ to A_G4_ and B_iM_.(*k_3_*); ΔH_4_ refers to the enthalpy change accompanying the monomolecular unfolding process of the central domain in duplex form – transition of species AB_wc_ to AB (*k_4_*).

- **G33/C33*Ff - mutually exclusivity model**

1. A *^k^_1_* A_G4_ $k1= \frac{[AG4]}{[A]}$
2. AB  *^k^_1_* A_G4_B $k1= \frac{[AG4B]}{[AB]}$
3. B *^k^_2_* B_iM_ $k2= \frac{[BiM]}{[B]}$
4. AB *^k^_2_* AB_iM_ $k2= \frac{[ABiM]}{[AB]}$
5. A + B *^k^_3_* AB $k3=\frac{[AB]}{\left[ A \right]*[B]}$
6. A_G4_ + B *^k^_3_* A_G4_B $k3= \frac{[AG4B]}{\left[ AG4 \right]*[B]}$
7. A + B_iM_ *^k^_3_*  AB_iM_ $k3= \frac{[ ABiM]}{\left[ BiM \right]*[B]}$

$Cp=\Delta H1 \frac{d\left( AG4 \right)}{d(T)}+\Delta H2 \frac{d(BiM)}{d(T)}$ + $\Delta H3 \frac{d(AB)}{d(T)}+(\Delta H1$+ $\Delta H3$) $\frac{d(AG4B)}{d(T)}$ $+(\Delta H2$+ $\Delta H3$) $\frac{d(ABiM)}{d(T)}$ (10)

where ΔH_1_ refers to the enthalpy change accompanying the G4 unfolding process - transitions of the species A_G4_ to A, and A_G4_B to AB (*k_1_*); ΔH_2_ refers to the enthalpy change accompanying the iM unfolding process - transitions of the species B_iM_ to B, and AB_iM_ to AB (*k_2_*); ΔH_3_ refers to the enthalpy change accompanying the unfolding of lateral duplexes – transition of species AB to A and B, A_G4_B to A_G4_ and B, and AB_iM_ to A and B_iM_, (*k_3_*);

**Reconstruction of CD melting profile of G33/C33*Ff from DSC-derived species distribution**

To derive CD melting from the species distribution derived from DSC data analyses CD spectra were obtained according to:

$R=M\cdot S(t)$ (11)

where R is the matrix of the predicted CD spectra, M is the matrix with the CD spectra measured at 20 °C of the species considered in the studied equilibrium (single-stranded and double-stranded constructs) reported in Δε (M^-1^ cm^-1^), and S(t) is the transposed matrix of the species fraction (from 0 to 1 values) upon increasing temperatures obtained through DSC fitting.

In these analyses, ΔH_i_ was considered as a temperature-independent parameter (heat capacity change is neglected for each transition). Data analysis, including fitting of the model functions (eqs. 3 -10) to experimental data, was performed using NumPy and SciPy Python libraries. Parameter errors are presented as standard deviations of the best-fit thermodynamic parameters obtained by fitting the corresponding model function to three consecutive DSC scans.

**Native Polyacrylamide Gel Electrophoresis (PAGE)**

DNA samples at the required concentrations were annealed in cacodylate buffer at appropriate pH with either 50 mM KCl or LiCl. They were loaded on a 10% native polyacrylamide gel (acrylamide: bis-acrylamide 19:1) in 1x TAE buffer (40 mM Tris, 20 mM acetic acid, 1 mM EDTA, pH 5.0) with 20 mM KCl. Gels were run at 25°C for 4 hours, by applying a voltage of 6.25 V/cm. Then, they were stained with a 1X Sybr Green II solution and visualized by an Invitrogen iBright imager (ThermoFisher).

**Thermal Difference Spectra (TDS)**

UV-Vis experiments were conducted on a Jasco J-815 spectropolarimeter equipped with a Peltier accessory for temperature control, using a quartz cuvette with either 0.1 cm or 1 cm path length. Oligonucleotide samples were prepared in the same conditions used for CD experiments. TDS corresponds to the arithmetic difference between the UV spectra recorded under heating conditions (unfolded structure) and cooling conditions (folded structure)^[64]^.

**NMR spectroscopy**

^1^H NMR samples were prepared at 80 μM DNA concentration in cacodylate buffer, pH 5.0, 50 mM KCl with 10% D_2_O. Prior to data acquisition, oligonucleotide solutions were annealed by heating to 95 °C for 5 min, followed by slow cooling to room temperature overnight. ^1^H NMR spectra were recorded at 25 °C on a Bruker DMX 600 MHz spectrometer equipped with a 5 mm TXI probe with XYZ-Gradient. For each spectrum, 2048 scans were acquired. Water suppression was achieved using the excitation sculpting pulse sequence. NMR spectra were processed and analysed using TopSpin.

**Small Angle X-ray Scattering (SAXS)**

DNA samples for SAXS measurements were prepared in cacodylate buffer, pH 5.0, 50 mM KCl at the following concentration: G33, G22, C33 sequences at 90 μM strand concentration; G33F, G33mF, C33*f and C33mf at 40 µM strand concentration; G33Ff, G33mFf, C33*Ff and C33mFf constructs at 25 μM oligo concentration; G33/C33Ff, G33/C33mFf, G33m/C33*Ff and G33/C33*Ff and G33m/C33mFf constructs at 25 μM oligo concentrations.

Small-angle X-ray scattering (SAXS) experiments were performed at the BM29 beamline of the European Synchrotron Radiation Facility (ESRF) in Grenoble, France. The incident energy was 12.5 keV, corresponding to an incident wavelength of 0.99 Å^–1^. All patterns were collected at 20 °C. Analogous patterns of the buffer were collected before and after every collection of the samples and used to subtract any contribution from the solvent and the sample environment.

The radius of gyration (Rg) and the forward scattering intensity (I(0)) were determined using the Guinier approximation^[65]^.

**Supporting Figures**


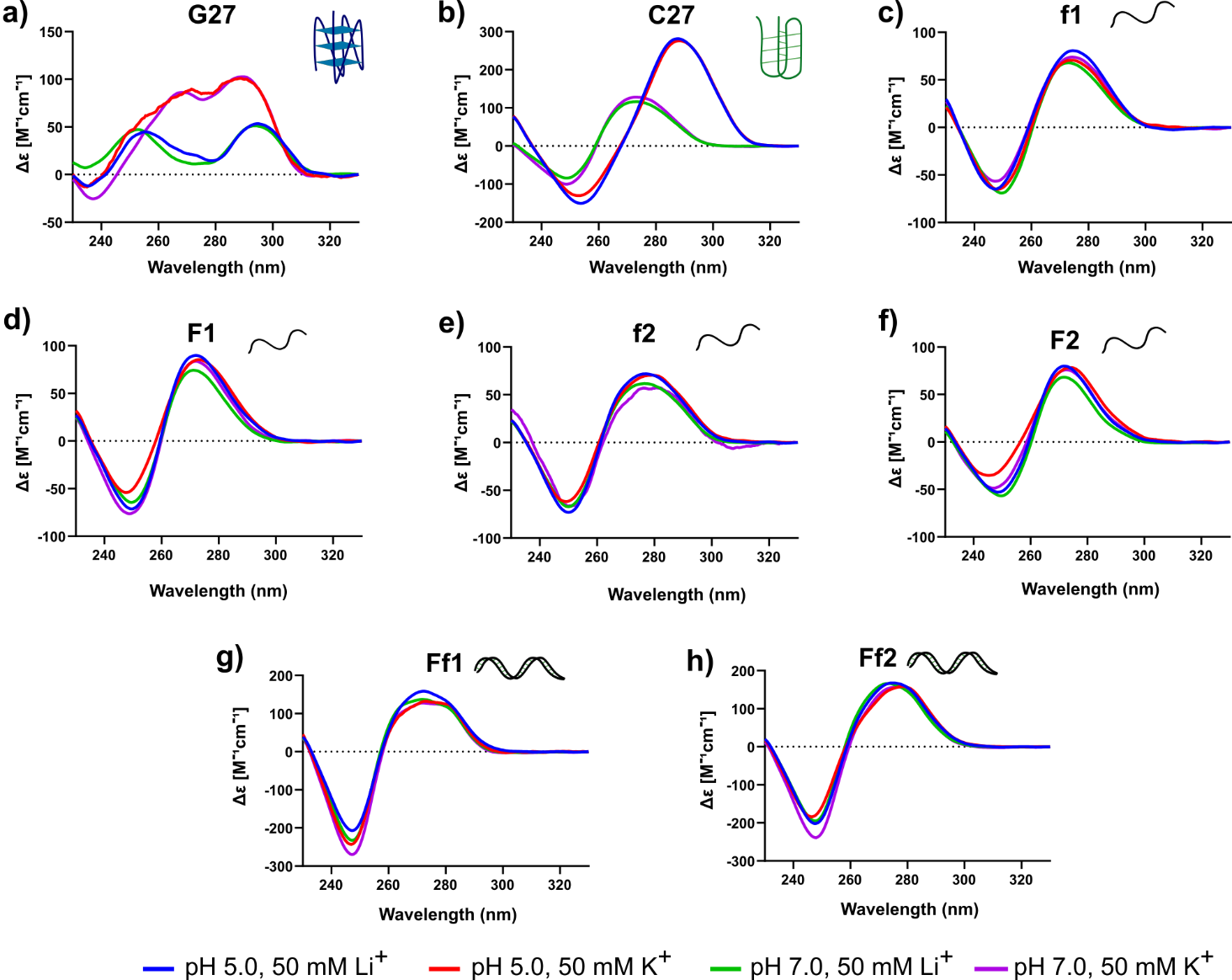


**Figure S1.** CD spectra acquired in cacodylate buffer, pH 7.0, with 50 mM KCl (purple lines) or 50 mM LiCl (green lines) and at pH 5.0, with 50 mM KCl (red lines) or 50 mM LiCl (blue lines) for the building block 3 µM G27(a), C27(b), f1(c), F1(d), f2(e), F2(f), Ff1 (g) and Ff2 (h).


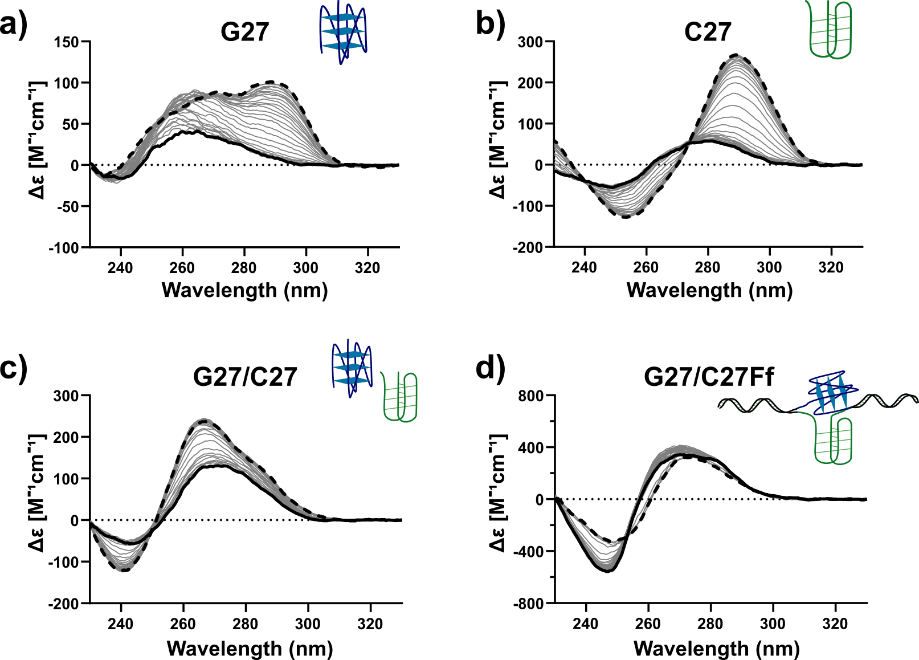


**Figure S2.** CD spectra recorded upon increasing temperature of a) 3 μM G27, b) 3 μM C27, c) 30 μM G27/C27 and d) 10 μM G27/C27 acquired in cacodylate buffer, pH 5.0, 50 mM KCl.


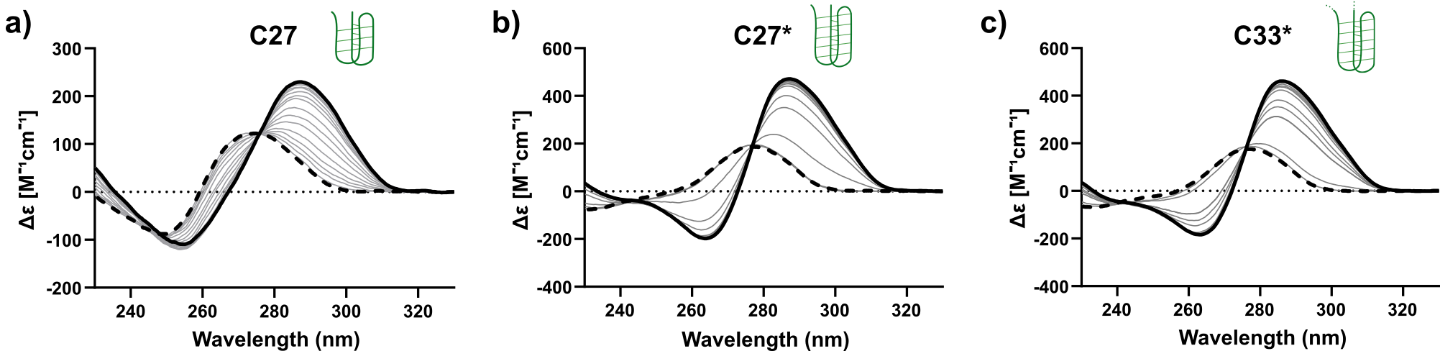


**Figure S3.** pH titration of the tested iM modules: CD spectra of 3 μM a) C27, b) C27* and c) C33* in 50 mM cacodylic acid (dashed line) upon increasing LiOH concentration.

**
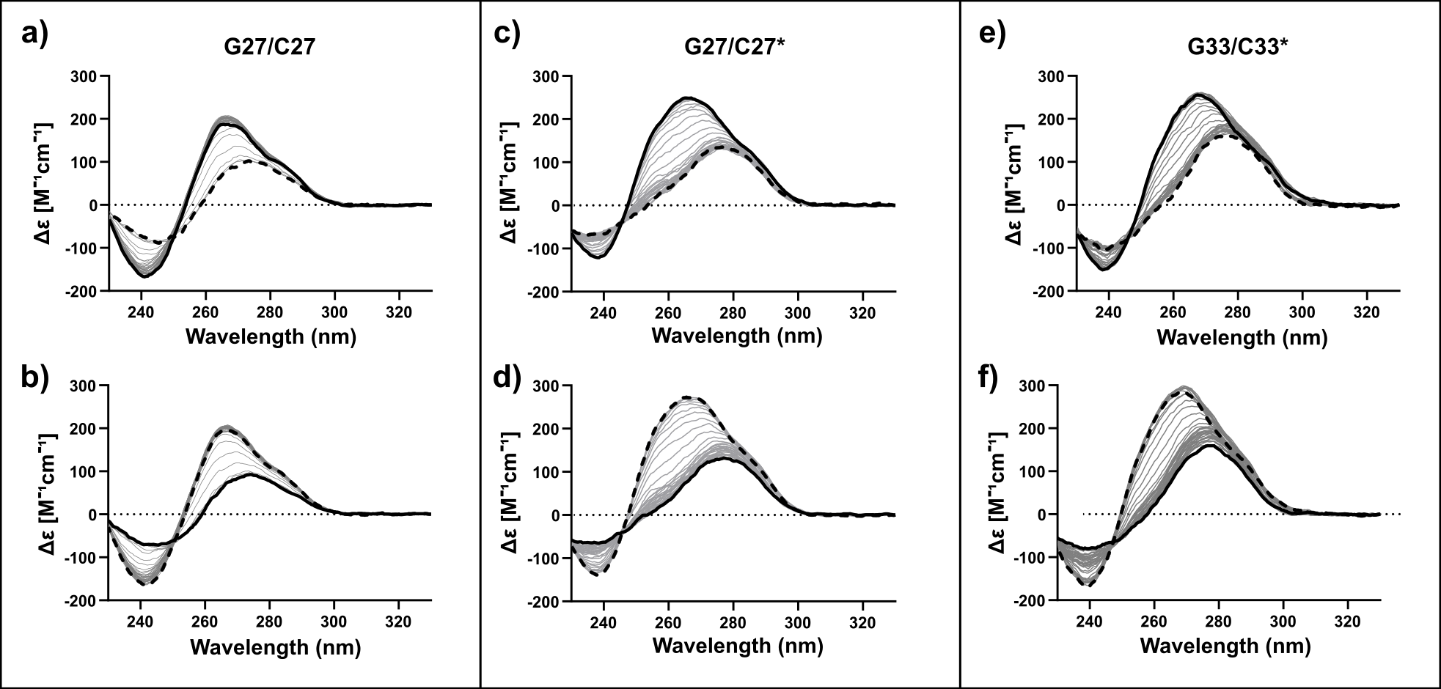
**

**Figure S4.** CD melting (a), c) and e)) and annealing (b), d) and f)) acquired in cacodylate buffer, pH 7.5, 50 mM KCl of 30 μM a-b) G27/C27, c-d) G27/C27* and e-f) G33/C33*.


**Figure S5**. Thermal Difference Spectra (TDS) of 4 μM G33 (blue line) and G27 (red line), in cacodylate buffer, pH 5.0, 50 mM KCl.


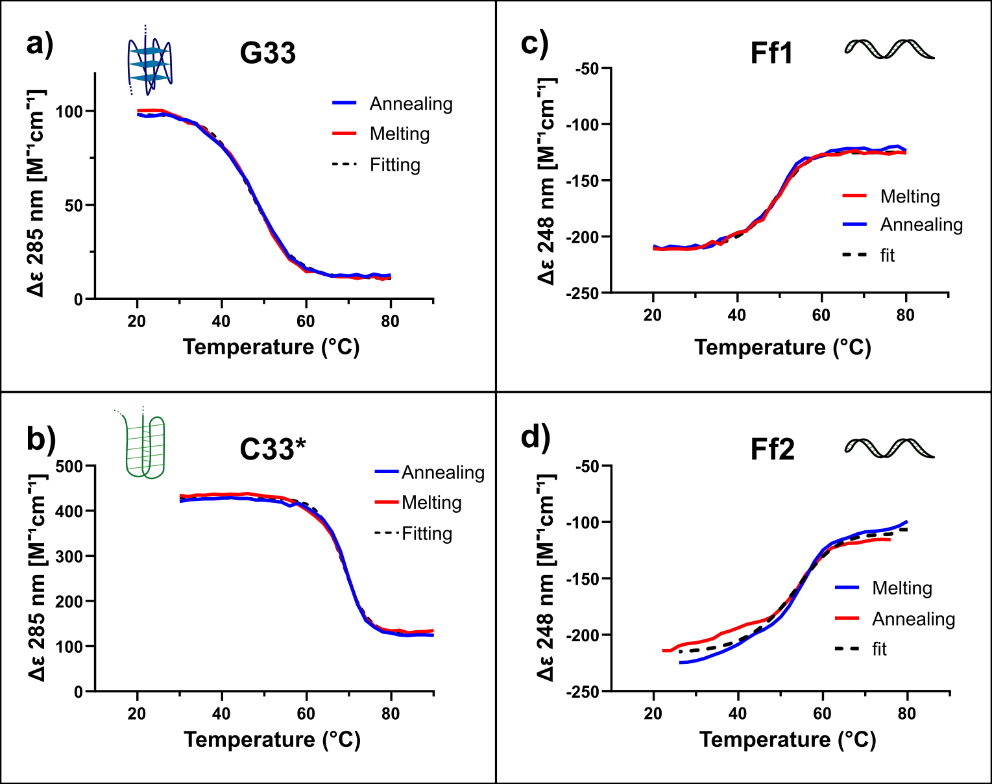


**Figure S6**. Variation of CD signals as a function of the temperature for a) 3 μM G33, b) 3 μM C33*, c) 30 μM Ff1 and d) 30 μM Ff2 recorded at selected wavelengths (290, 285 and 248 nm) in cacodylate buffer, pH 5.0, 50 mM KCl. Red and blue lines refer to melting and annealing processes, respectively. Black dashed lines correspond to the fitting curves according to Eq.1 for G33 and C33* and Eq.2 for Ff1 and Ff2.


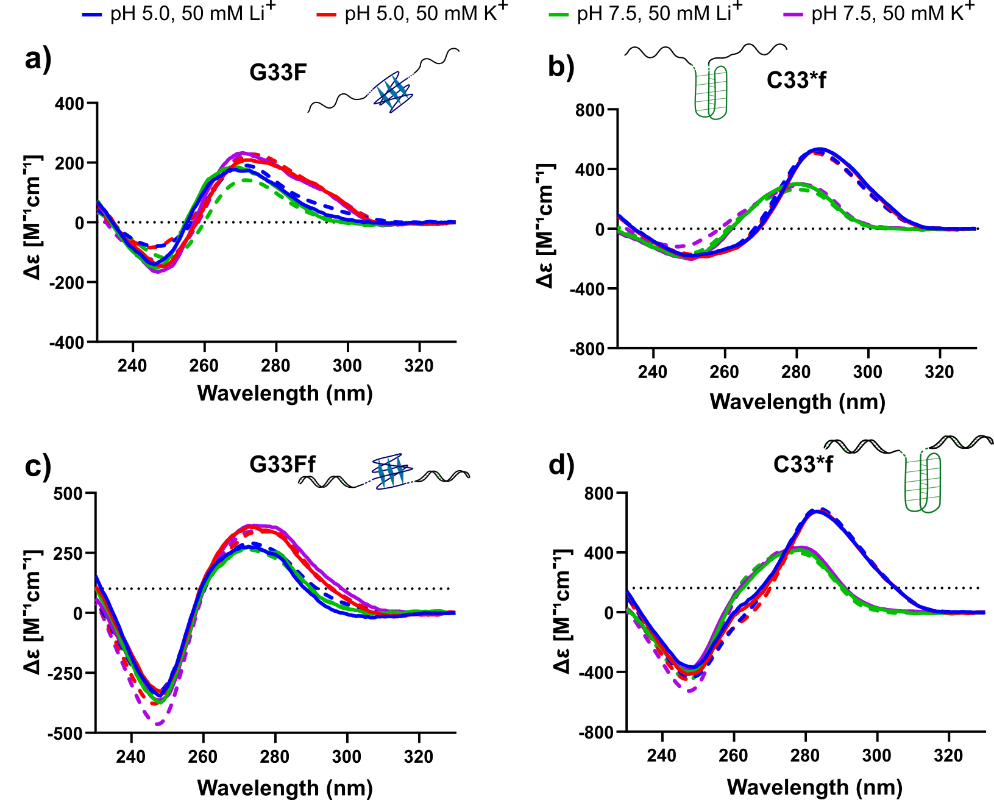


**Figure S7**. Comparison of the experimentally acquired CD spectra (solid lines) with the linear combination obtained from the reference spectra reported in Figure S1 (dotted lines) for 1 µM a) G33F, b) C33*f, c) G33Ff, d) C33*Ff in cacodylate buffer at pH 7.5, with 50 mM KCl (purple lines) or 50 mM LiCl (green lines) and at pH 5.0, with 50 mM KCl (red lines) or 50 mM LiCl (blue lines)


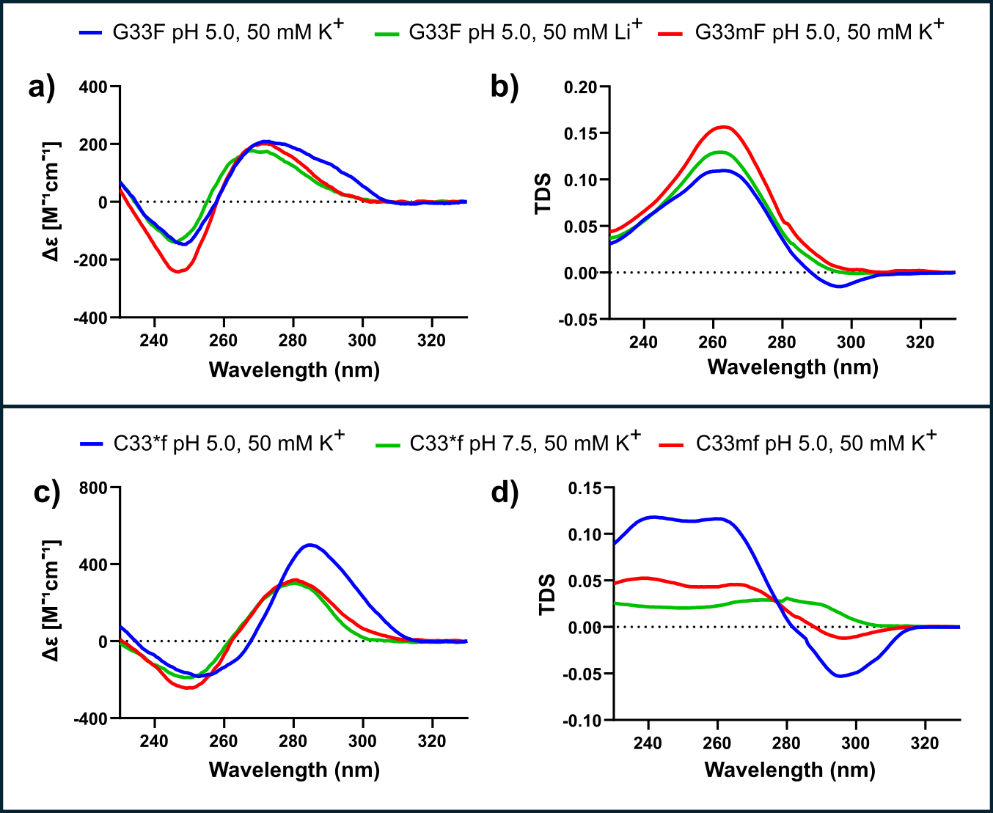


**Figure S8**. a) CD and b) TDS spectra of 1 µM G33F acquired in cacodylate buffer at pH 5.0, with 50 mM KCl (blue line) or 50 mM LiCl (green line) and 1 µM G33mF at pH 5.0, 50 mM KCl (red line); c) CD spectra and d) TDS spectra of 1 µM C33*f acquired in cacodylate buffer, at pH 5.0, 50 mM KCl (blue line), at pH 7.5, 50 mM KCl (green line) and 1 µM C33mf at pH 5.0, 50 mM KCl (red line);


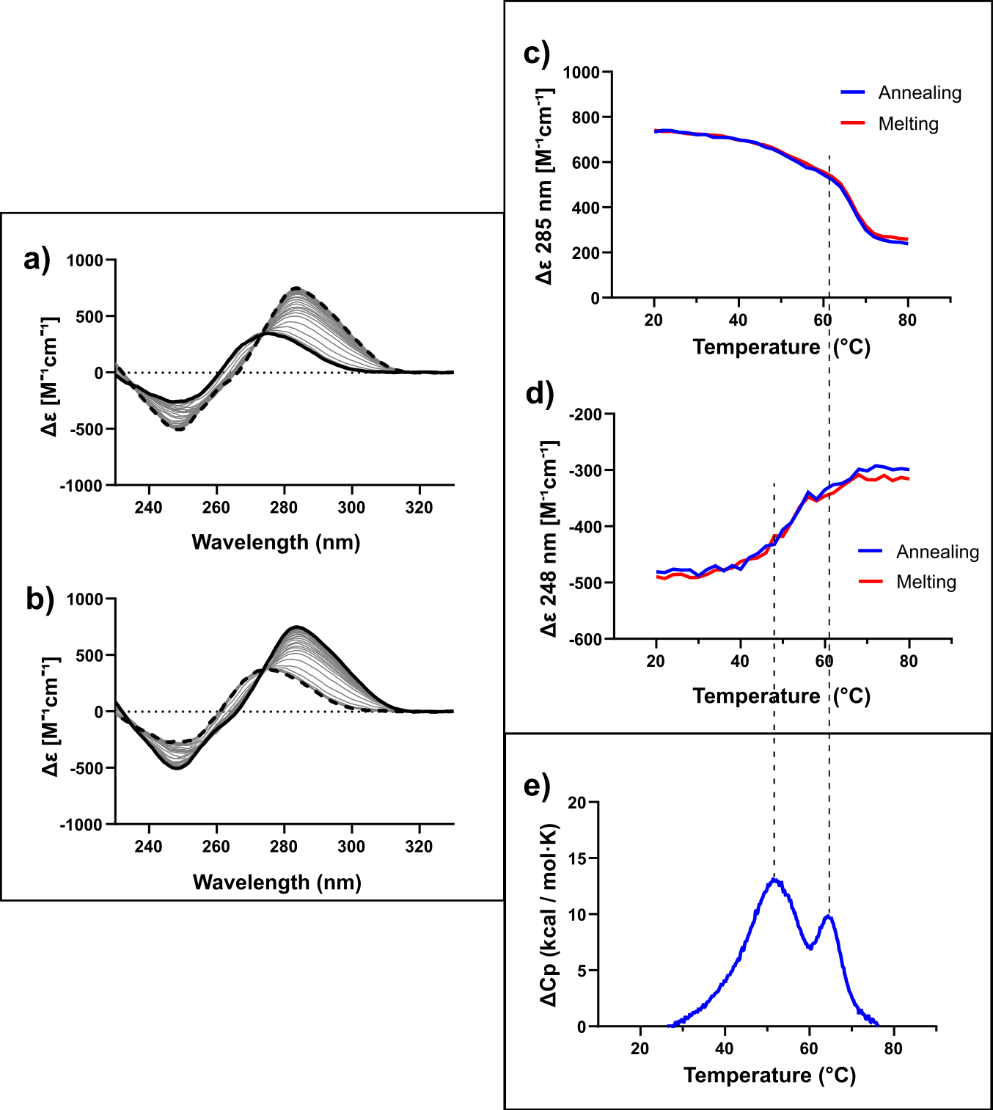


**Figure S9.** CD spectra acquired during the a) melting and b) annealing of 10 μM G33/C33*Ff in cacodylate buffer at pH 5.0, 50 mM KCl and corresponding variations of the CD signal recorded at c) 285 nm and d) 248 nm (red and blue lines for melting and annealing, respectively); e) DSC thermogram of 30 μM G33/33*Ff acquired in cacodylate buffer, pH 5.0, 50 mM KCl. Dotted lines highlight the alignment of the CD and DSC transitions.


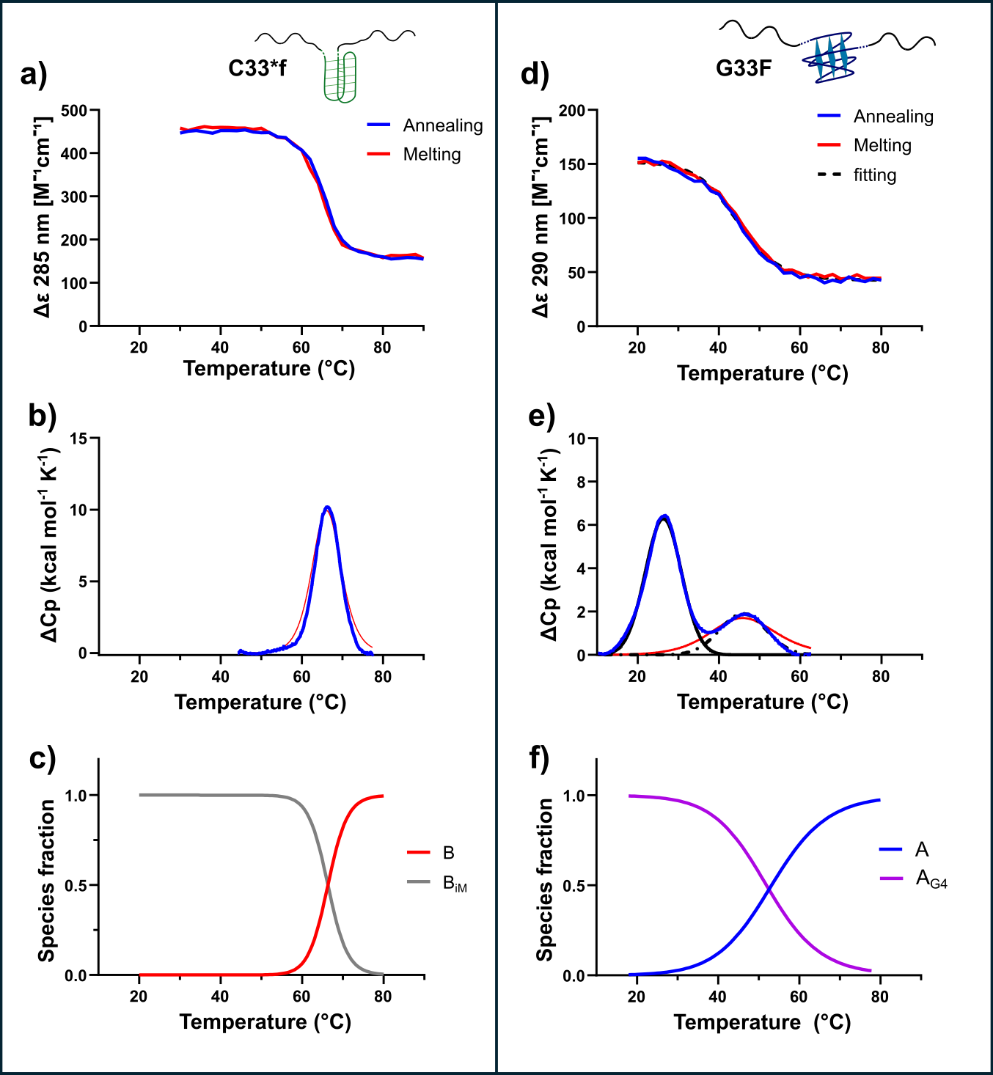


**Figure S10**. a) Variation of the CD signal upon melting (red line) and annealing (blue line) processes for 1 μM C33*f, recorded at 285 nm in cacodylate buffer, pH 5.0, 50 mM KCl, fitted according to Eq.(1) (black dashed line) b) DSC thermogram (blue line) of 150 μM C33*f in cacodylate buffer, pH 5.0, 50 mM KCl, fitted according to Eq.(3) (red line); c) derived species distribution; d) Variation of the CD signal upon melting (red line) and annealing (blue line) processes for 1 μM G33F, recorded at 290 nm in cacodylate buffer, pH 5.0, 50 mM KCl, fitted according to Eq.(1) (black dashed line); e) DSC thermogram (blue line) of 250 μM G33F in cacodylate buffer, pH 5.0, 50 mM KCl, deconvoluted in two main peaks (black lines) and fitting of the higher temperature peak according to Eq.(4) (red line); f) derived species distribution.


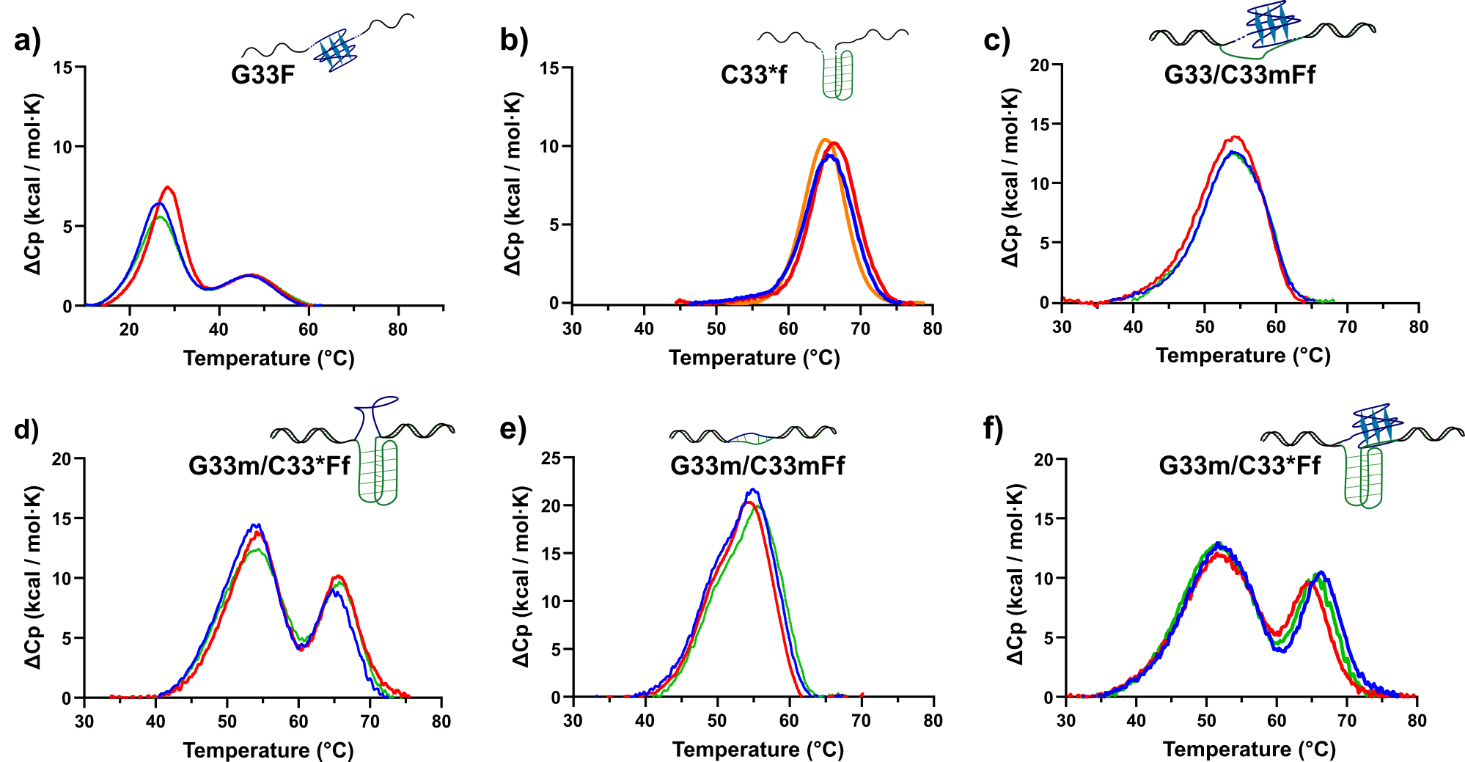


**Figure S11**. Overlay of three consecutive melting-annealing-melting DSC scans of a) 250 µM G33F, b) 150 µM C33*f, c) 30 µM G33/C33mFf, d) 30 µM G33m/C33*Ff, e) 30 µM G33m/C33mFf and f) 30 µM G33/C33*Ff in cacodylate buffer, pH 5.0, 50 mM KCl.


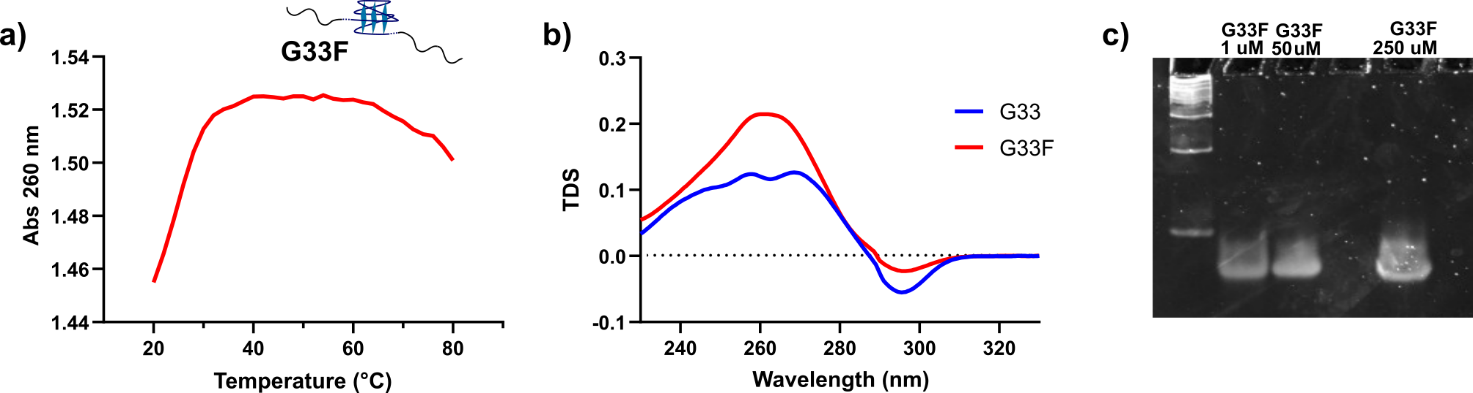


**Figure S12**. a) Variation of the UV signal as a function of temperature for 1 μM G33F recorded at 260 nm in cacodylate buffer, pH 5.0, 50 mM KCl; b) TDS spectra of 1 μM G33 (blue line) and G33F (red line) acquired in cacodylate buffer, pH 5.0, 50 mM KCl; c) Native PAGE of increasing concentration of G33F previously annealed in cacodylate buffer, pH 5.0, 50 mM KCl.

*
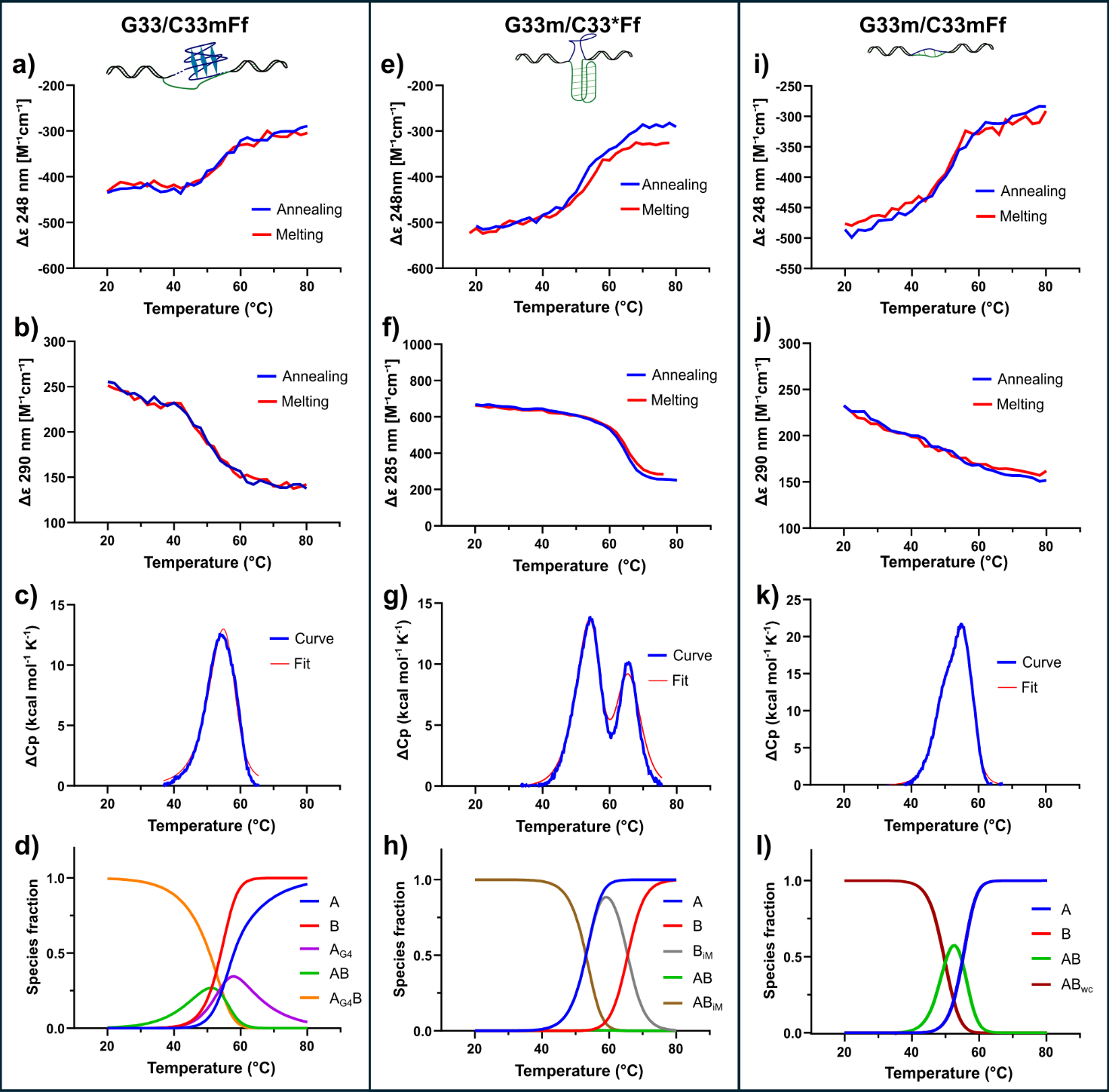
*

**Figure S13**. a)-d): Variation of the CD signal upon melting (red line) and annealing (blue line) processes of 10 μM G33/C33mFf recorded at 248 nm (a) and 290 nm (b) in cacodylate buffer, pH 5.0, 50 mM KCl; c) DSC thermogram (blue line) of 30 μM G33/C33mFf in cacodylate buffer, pH 5.0, 50 mM KCl, fitted according to Eq.(5) (red line) and d) the derived species distribution; e)-h): Variation of the CD signal upon melting (red line) and annealing (blue line) processes of 10 μM G33m/C33*Ff recorded at 248 nm (e) and 285 nm (f) in cacodylate buffer pH 5.0, 50 mM KCl; g) DSC thermogram (blue line) of 30 μM G33m/C33*Ff in cacodylate buffer, pH 5.0, 50 mM KCl, fitted according to Eq.(6) (red line) and h) the derived species distribution; i)-l): Variation of the CD signal upon melting (red line) and annealing (blue line) processes of 10 μM G33m/C33mFf recorded at 248 nm (i) and 290 nm (j) in cacodylate buffer, pH 5.0, 50 mM KCl; k) DSC thermogram (blue line) of 30 μM G33m/C33mFf in cacodylate buffer, pH 5.0, 50 mM KCl, fitted according to Eq.(7) (red line) and l) the derived species distribution.


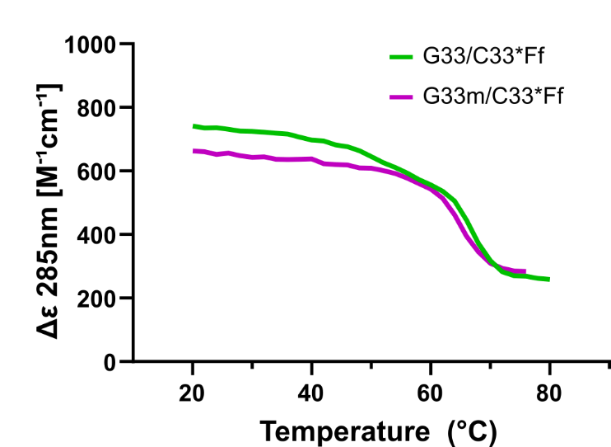


**Figure S14.** Variation of CD signals as a function of temperature of 10 μM G33F/C33*Ff (green line) and G33m/C33*Ff (violet line) recorded at 285 nm in cacodylate buffer, pH 5.0, 50 mM KCl.

*
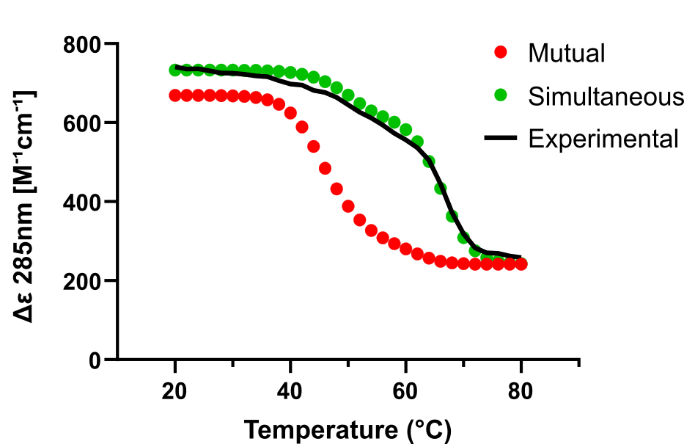
*

**Figure S15.** Comparison of the variation of CD signals as a function of temperature of G33F/C33*Ff experimentally acquired (black line) recorded at 285 nm in cacodylate buffer, pH 5.0, 50 mM KCl, to those predicted from the parameters derived according to the G4/iM simultaneous (Eq.8, green dots) and mutually exclusive model (Eq.10, red dots).

**
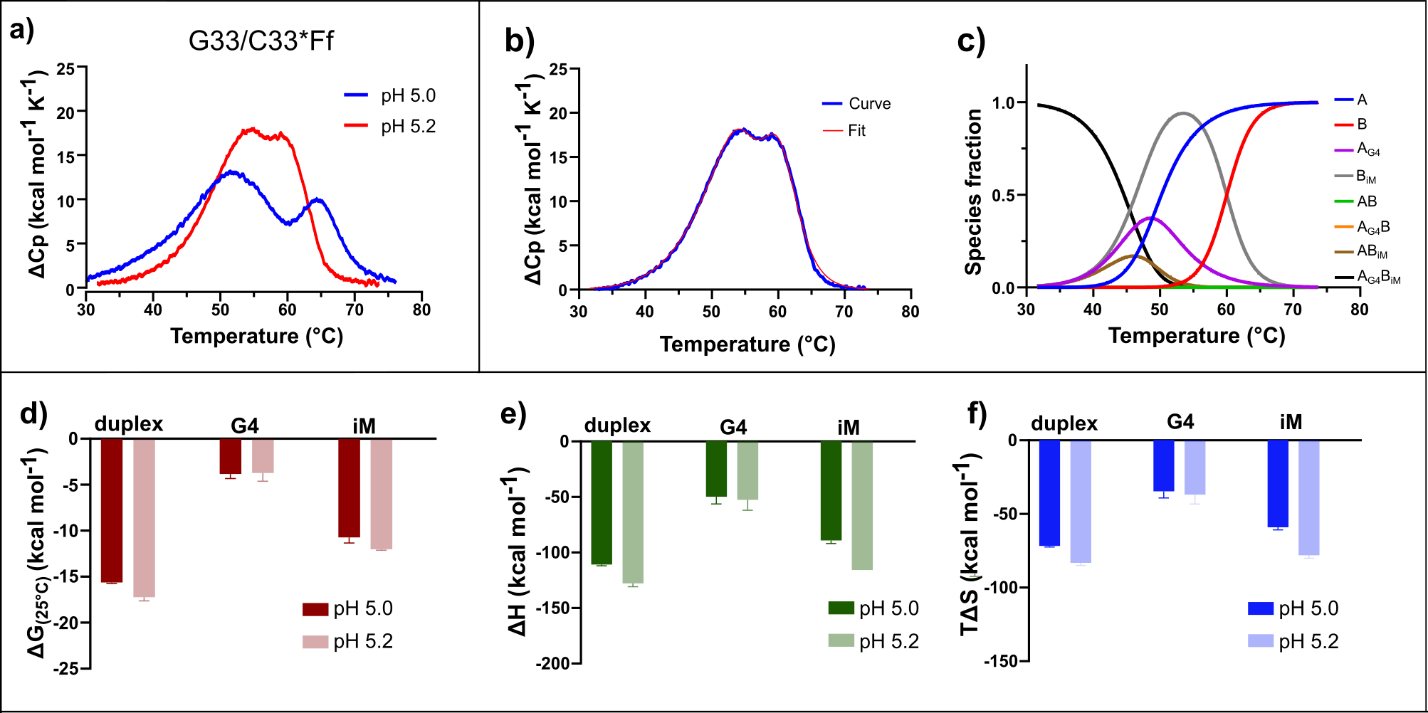
**

**Figure S16.** Characterization of thermodynamic equilibria at different pH.a) DSC thermograms of 30 μM G33/C33*Ff construct acquired in cacodylate buffer,50 mM KCl at pH 5.0 (blue line) and pH 5.2 (red line); b) DSC thermogram of G33/C33*Ff construct at pH 5.2 (blue line) fitted with Eq. 8, from which we calculated the species fraction (c); d) ΔG, e) ΔHs, f) TΔS values of distinct structural domains calculated by fitting the DSC thermograms of G33/C33*Ff acquired in cacodylate buffer, 50 mM KCl at pH 5.0 and 5.2 according to eq. (8).

**
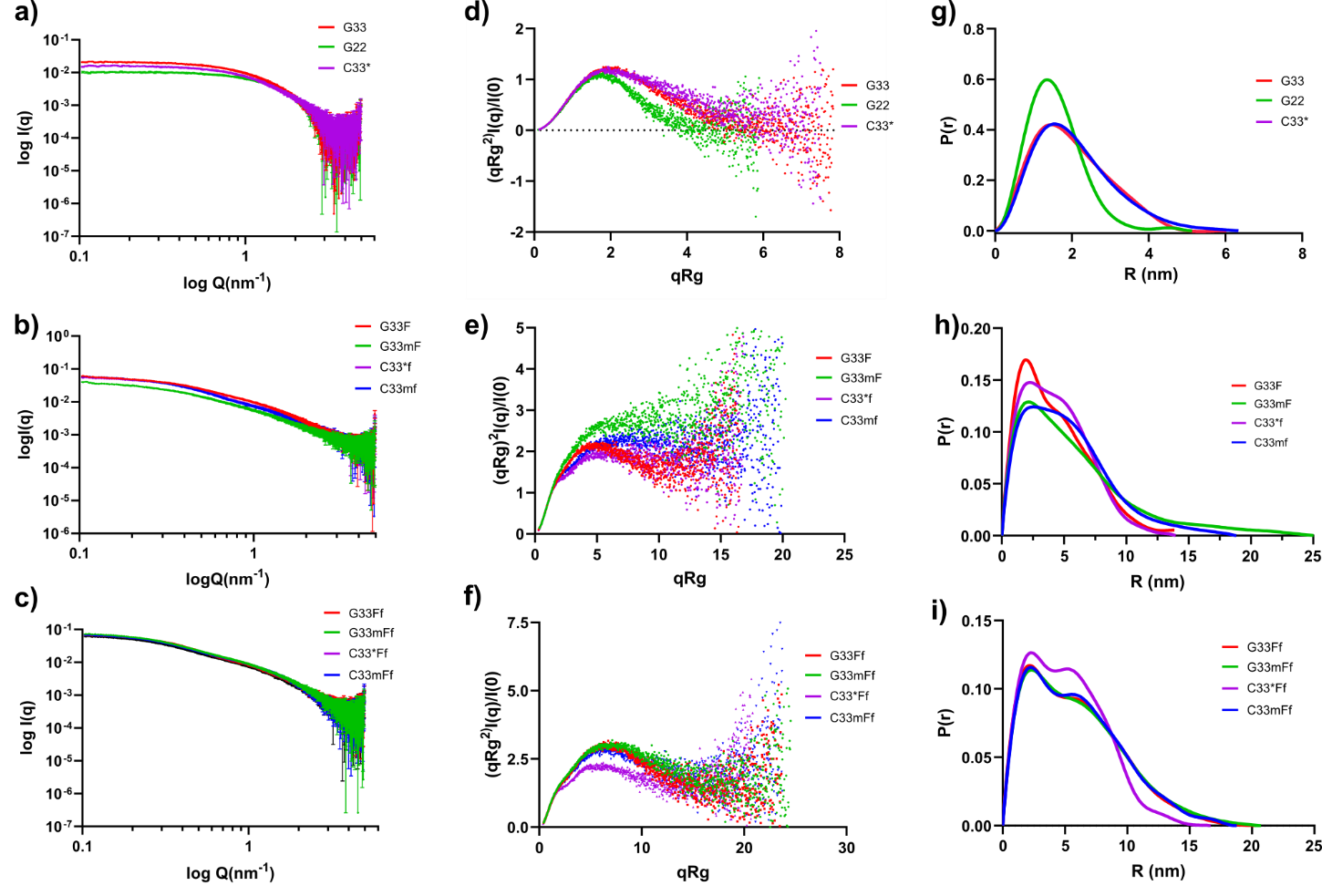
**

**Figure S17** SAXS data acquired in cacodylate buffer, pH 5.0, 50 mM KCl .a) Intensity data, d) Kratky Plot and g) P(r) function of 90 µM G33 (red line), G22 (green line), C33* (violet line); b) Intensity data, e) Kratky Plot and h) P(r) function of 40 µM G33F (red line), G33mF (green line), C33*f (violet line) and C33mf (blue line) sequences; c) Intensity data, f) Kratky Plot and i) P(r) function of 25 µM G33Ff (red line), G33mFf (green line), C33*Ff (violet line), C33mFf (blue line) constructs;

**Supporting Tables**

**Table S1**. Name and composition of the DNA sequences used in this study

| **Name** | **Sequence (5’-3’)** |
| --- | --- |
| **F1** | CAA TCC AGA TAT ATT TAA TAA |
| **F2** | ATA TTA TAT TTA GCA GAG ACG |
| **f1** | TTA TTA AAT ATA TCT GGA TTG |
| **f2** | CGT CTC TGC TAA ATA TAA TAT |
| **G27** | TTA GGG TTA GGG TTA GGG TTA GGG TTA |
| **G33** | TTA TTA GGG TTA GGG TTA GGG TTA GGG TTA TTA |
| **G33m** | TTA TTA GGG TTA CCG TTA GCC TTA GGG TTA TTA |
| **C27** | TAA CCC TAA CCC TAA CCC TAA CCC TAA |
| **C27*** | CCC CTT TCC CCC TTT CCC CTT TCC CCC |
| **C33*** | TTT CCC CTT TCC CCC TTT CCC CTT TCC CCC TTT |
| **C33m** | TTT CCC CTT TCC GGC TTT CGG CTT TCC CCC TTT |
| **G27F** | CAA TCC AGA TAT ATT TAA TAA TTA GGG TTA GGG TTA GGG TTA GGG TTA ATA TTA TAT TTA GCA GAG ACG |
| **G33F** | CAA TCC AGA TAT ATT TAA TAA TTA TTA GGG TTA GGG TTA GGG TTA GGG TTA TTA ATA TTA TAT TTA GCA GAG ACG |
| **G33mF** | CAA TCC AGA TAT ATT TAA TAA TTA TTA GGG TTA CCG TTA GCC TTA GGG TTA TTA ATA TTA TAT TTA GCA GAG ACG |
| **C27f** | CGT CTC TGC TAA ATA TAA TAT TAA CCC TAA CCC TAA CCC TAA CCC TAA TTA TTA AAT ATA TCT GGA TTG |
| **C27*f** | CGT CTC TGC TAA ATA TAA TAT CCC CTT TCC CCC TTT CCC CTT TCC CCC TTA TTA AAT ATA TCT GGA TTG |
| **C33f** | CGT CTC TGC TAA ATA TAA TAT TAA TAA CCC TAA CCC TAA CCC TAA CCC TAA TAA TTA TTA AAT ATA TCT GGA TTG |
| **C33*f** | CGT CTC TGC TAA ATA TAA TAT TTT CCC CTT TCC CCC TTT CCC CTT TCC CCC TTT TTA TTA AAT ATA TCT GGA TTG |
| **C33mf** | CGT CTC TGC TAA ATA TAA TAT TTT CCC CTT TCC GGC TTT CGG CTT TCC CCC TTT TTA TTA AAT ATA TCT GGA TTG |
| **G22** | AGG GTT AGG GTT AGG GTT AGG G |

**Table S2.** Standard thermodynamic parameters of folding of G4, iM, and ds domains forming at minimal G33, C33*, Ff1, and Ff2 modules derived from analyses of CD melting annealing experiments acquired in cacodylate buffer, pH 5.0, 50 mM KCl, according to Equations (1) and (2). T_m_ referring to 1 M oligo concentration.

| Sequence | Structural Domain | dataset | ΔH (kcal mol^-1^) | ΔS(cal mol^-1^ K^-1^) | Tm (°C) | Concentration (µM) |
| --- | --- | --- | --- | --- | --- | --- |
| G33 | G4 | CD | -41.7 ± 0.9 | -130.1 ± 0.1 | 47.3 ± 0.1 | 3 |
| C33* | iM | CD | -81.3 ± 2.8 | -237.9 ± 0.3 | 68.8 ± 0.4 | 3 |
| Ff1 | ds | CD | -81.3 ± 7.5 | -228.9 ± 24.2 | 82.2 ± 5.2 | 30 |
| Ff2 | ds | CD | -65.3 ± 9.8 | -174.1 ± 30.2 | 102.9 ± 7.6 | 30 |

**Table S3.** Melting Temperature (T_m_) of G4, iM and ds domains forming at minimal (G33, C33*, Ff1, and Ff2), full length single (G33F, C33f) and double stranded (G33/C33mFf, G33m/C33*Ff, G33m/C33mFf) working models derived from the analyses of CD and/or DSC melting/annealing profiles acquired in cacodylate buffer, pH 5.0, 50 mM KCl. T_m_ referring to 1 M construct concentration.

| Species | Sequence | Structural  Domain | T_m_ (°C)  CD | T_m_ (°C)  DSC | Concentration (µM) |
| --- | --- | --- | --- | --- | --- |
| A_G4_ | G33F | G4 | 44.6 ± 0.1 | 46.8 ± 0.5 | 1 (CD)  250 (DSC) |
| B_iM_ | C33*f | iM | 64.9 ± 0.1 | 65.6 ± 0.6 | 1 (CD)  150 (DSC) |
| A_G4_B | G33/C33mFf | ds |  | 74.2 ± 0.3 | 30 |
|  |  | G4 |  | 51.5 ± 4.4 |  |
| AB_iM_ | G33m/C33*Ff | ds |  | 72.0 ± 0.7 | 30 |
|  |  | iM |  | 65.1 ± 0.3 |  |
| AB_wc_ | G33m/C33mFf | ds |  | 72.0 ± 0.9 | 30 |
|  |  | central pairing |  | 50.4 ± 0.5^[a]^ |  |

**Table S4.** Standard thermodynamic parameters of folding of G4, iM and ds domains within the full-length construct G33/C33*Ff derived by fitting of the model function (eq. 9) to DSC thermograms acquired in cacodylate buffer, pH 5.0, with 50 mM KCl.

| Construct | Structural Domain | ΔH  (kcal mol⁻¹) | ΔS  (cal mol^-1^ K^-1^) | ΔG_(298.15 K)_  (kcal mol⁻¹) |
| --- | --- | --- | --- | --- |
| G33/C33*Ff | ds flankings | -107.2 ± 1.6 | -308.5 ± 4.9 | -15.2 ± 0.2 |
|  | G4 | -54.3 ± 5.1 | -168.3 ± 15.8 | -4.1 ± 0.4 |
|  | iM | -89.4 ± 2.4 | -264.1 ± 6.6 | -10.7 ± 0.5 |
|  | Central pairing | -57.1 ± 7.0 | -191.6 ± 22.2 | -0.1 ± 0.5 |

**Table S5.** Melting Temperature (T_m_) of G4, iM and ds domains within the full-length construct G33/C33*Ff derived by the fitting DSC thermograms acquired in cacodylate buffer, pH 5.0, 50 mM KCl, according to the reported equations and referring to 1 M construct concentration.

| Equation | Structural  Domain | T_m_ (°C) |
| --- | --- | --- |
| (8) | ds flankings | 73.7 ± 0.3 |
|  | G4 | 49.1 ± 0.4 |
|  | iM | 65.3 ± 1.0 |
| (9) | ds flankings | 74.4 ± 0.3 |
|  | G4 | 49.6 ± 0.2 |
|  | iM | 65.3 ± 0.8 |
|  | Central pairing | 24.8 ± 2.6 |
| (9)^[a]^ | ds flankings | 74.4 ± 0.1 |
|  | G4 | 49.6 ± 0.2 |
|  | iM | 65.3 ± 1.0 |
| (10) | ds flankings | 86.7 ± 1.0 |
|  | G4 | 52.7 ± 0.3 |
|  | iM | 49.2 ± 0.4 |

[a] Calculated using ΔH_4_ = - 80.0 kcal mol ^-1^ and T_m4_ = 50 °C as constrains

**Table S6.** Standard thermodynamic parameters of folding of G4, iM and ds domains within the full-length construct G33/C33*Ff derived by the fitting of DSC thermograms acquired in cacodylate buffer, pH 5.2, 50 mM KCl, according to Eq. (8). T_m_ refers to 1 M construct concentration.

| pH | Structural Domain | ΔH  (kcal mol^-1^) | ΔS  (cal mol^-1^ K^-1^) | ΔG_(298.15 K)_  (kcal mol^-1^) | T_m_  (°C) |
| --- | --- | --- | --- | --- | --- |
| 5.2 | ds flankings | -128.3 ± 2.9 | -372.6 ± 8.2 | -17.2 ± 0.4 | 71.2 ± 0.2 |
|  | G4 | -53.1 ± 9.4 | -165.7 ± 28.8 | -3.7 ± 0.9 | 47.4 ± 1.8 |
|  | iM | -116.3 ± 2.7 | -349.8 ± 8.8 | -12.0 ± 0.1 | 59.3 ± 0.7 |

**Table S7**. Model-independent analysis of small-angle X-ray scattering data of tested sequences previously annealed in cacodylate buffer, pH 5.0, 50 mM KCl.

| Sequence | Molecular weight  (g mol^-1^) | I(0) | R_g_ (nm) | D_max_ (nm) |
| --- | --- | --- | --- | --- |
| G22 | 6957.5 | 0.011 | 1.19 ± 0.01 | 3.82 |
| G33 | 10339.7 | 0.021 | 1.58 ± 0.01 | 4.94 |
| C33* | 9706.3 | 0.016 | 1.52 ± 0.01 | 4.81 |
| G33F | 23328.2 | 0.056 | 3.34 ± 0.02 | 13.82 |
| G33mF | 23168.1 | 0.039 | 4.09 ± 0.04 | 22.73 |
| C33*f | 22658.7 | 0.053 | 3.40 ± 0.02 | 12.54 |
| C33mf | 22818.8 | 0.061 | 4.04 ± 0.03 | 17.50 |
| G33Ff | 36156.7 | 0.073 | 4.79 ± 0.07 | 17.03 |
| G33mFf | 35996.6 | 0.078 | 4.96 ± 0.08 | 17.89 |
| C33*Ff | 35523.3 | 0.070 | 4.20 ± 0.07 | 14.23 |
| C33mFf | 35683.4 | 0.066 | 4.81 ± 0.07 | 17.48 |
| G33/C33mFf | 46147.0 | 0.140 | 5.16 ± 0.02 | 19.41 |
| G33m/C33*Ff | 45826.8 | 0.150 | 4.32 ± 0.01 | 16.00 |
| G33m/C33mFf | 45986.9 | 0.140 | 5.33 ± 0.03 | 20.47 |
| G33/C33*Ff | 45986.9 | 0.130 | 4.70 ± 0.02 | 18.22 |
| G33/C33Ff | 46203.2 | 0.130 | 6.04 ± 0.05 | 22.73 |

**Table S8**. Model-independent analysis of small-angle X-ray scattering data of different pairs of tested sequences annealed under conditions that prevent the formation of non-canonical secondary structures in the central domain (cacodylate buffer at different pH with 50 mM LiCl or KCl).

| Sequence | Molecular weight  (g mol^-1^) | I(0) | R_g_ (nm) | D_max_ (nm) |
| --- | --- | --- | --- | --- |
| Gm33/C33mFf  pH 5.0, 50 mM KCl | 45986.9 | 0.140 | 5.33 ± 0.03 | 20.47 |
| G33/C33mFf  pH 5.0, 50 mM LiCl | 46147.0 | 0.130 | 5.25 ± 0.02 | 20.14 |
| G33m/C33*Ff  pH 7.5, 50 mM KCl | 45826.8 | 0.140 | 5.49 ± 0.03 | 20.67 |
| G33/C33Ff  pH 5.0, 50 mM KCl | 46203.2 | 0.130 | 6.04 ± 0.05 | 22.73 |

**References**

[64] J.-L. Mergny, L. Lacroix, “Analysis of thermal melting curves” *Oligonucleotides* **2003**, *13*, 515–537.

[65] T. V. Chalikian, L. Liu, Jr. Macgregor Robert B., “Duplex-tetraplex equilibria in guanine- and cytosine-rich DNA” *Biophysical Chemistry* **2020**, *267*, 106473.

[66] J.-L. Mergny, J. Li, L. Lacroix, S. Amrane, J. B. Chaires, “Thermal difference spectra: a specific signature for nucleic acid structures” *Nucleic Acids Res* **2005**, *33*, e138.

[67] T. W. Gräwert, D. I. Svergun, “Structural Modeling Using Solution Small-Angle X-ray Scattering (SAXS)” *Journal of Molecular Biology* **2020**, *432*, 3078
